# Numerical Investigation of Downstream-Shaft Aeration and Air-Pocket Evolution in a Navigation-Lock Valve

**DOI:** 10.3390/e28090954

**Published:** 2026-08-25

**Authors:** Tingqiang Xie, Zhonghua Li, Xiujun Yan, Jun Deng, Duo Xu

**Affiliations:** 1 State Key Laboratory of Hydraulics and Mountain River Engineering, Sichuan University, Chengdu 610065, China; tqxie@nhri.cn (T.X.); djhao2002@scu.edu.cn (J.D.); 2 Hydraulic Engineering Department, Nanjing Hydraulic Research Institute, Nanjing 210029, China; xjyan@nhri.cn (X.Y.); xvduo@hhu.edu.cn (D.X.); 3 Key Laboratory of Navigation Structures Construction Technology, Nanjing Hydraulic Research Institute, Nanjing 210029, China

**Keywords:** filling-and-emptying valve, downstream-shaft aeration, entrapped air pocket, shear-layer instability

## Abstract

The filling-and-emptying valve and downstream shaft are crucial components of navigation-lock systems. Under insufficient downstream submergence, air can be drawn through the shaft and trapped in the post-valve culvert, altering the flow structure and compromising hydraulic stability. A three-dimensional Reynolds-averaged Navier–Stokes/volume-of-fluid model was developed to investigate shaft aeration and entrapped-air-pocket evolution under varying inlet velocities and downstream-submergence depths. The aeration process comprises three stages: jet establishment, air-pocket formation, and air-pocket breakup and reorganization. Downstream-submergence depth determines whether a continuous air-intake pathway forms, whereas inlet velocity primarily controls aeration intensity and air-pocket persistence once the pathway is established. With decreasing submergence depth, the flow transitions successively from a water-sealed regime to a transition regime, a stable entrapped-air-pocket regime, and a strongly unsteady hydraulic-jump-like regime. For the present geometry and fixed valve opening, the transition from transient to sustained shaft aeration is identified within the downstream-submergence interval of *h_w_* = 2–5 m. Combined analyses of the air-pocket volume per unit width, pressure response, vortex structures, and shear-layer characteristics indicate that enhanced jet-induced shear is closely associated with shaft aeration and air entrapment, while pressure fluctuations are closely coupled with air-pocket formation, persistence, breakup, and reorganization.

## 1. Introduction

The filling-and-emptying valve, hereafter referred to as the lock valve, is a key hydraulic structure in navigation-lock water conveyance systems. Vertical structures, such as downstream maintenance shafts and air vents, are commonly arranged downstream of the valve to meet the requirements of inspection, maintenance, ventilation, and air release. In this study, these vertical structures connected to the post-valve culvert are collectively referred to as the downstream shaft. Under the influence of extreme hydroclimatic events and river water-level variations, downstream water-level fluctuations in lock culverts have become more pronounced. During valve opening and closing, such fluctuations can induce pronounced local transient flows in the culvert, further leading to post-valve aeration in the downstream shaft, the formation of an entrapped air pocket, and even a strongly unsteady hydraulic jump [1]. When the entrapped air pocket interacts with the valve, culvert bends, or abrupt boundary transitions, intense impact loads may be generated and structural vibration may be induced; in severe cases, local structural failure may even occur, thereby threatening the operational efficiency and structural integrity of the lock system [2,3]. Therefore, post-valve aeration in the downstream shaft and the associated air–water two-phase flow characteristics constitute a significant hydraulic issue for the safe operation of navigation-lock filling-and-emptying systems.

Hydraulic issues associated with the lock valves in navigation locks have been systematically investigated in previous studies. Hu et al. [4] focused on low-pressure and cavitation problems in lock valve sections and proposed several improvement measures, including optimizing the culvert geometry, adopting variable speed valve-opening schedules, and arranging aeration structures at appropriate locations. With the development of computational fluid dynamics, numerical simulation has gradually become an important tool for studying valve hydraulic characteristics. Wan and Yu [5], Dai and Wei [6], and Ma et al. [7] investigated the flow characteristics in the valve-section culverts of the Three Gorges Lock. Scheffermann and Stockstill [8] calculated the pressure in the post-valve culvert, while Hammack and Stockstill [9] analyzed the flow pattern, pressure field, and discharge coefficient of the valve section under different valve openings. Yang et al. [10] optimized the geometry of the sudden expansion culvert downstream of a reverse tainter valve. Jiang et al. [11] focused on valve-lip cavitation in the valve section, optimized the valve-lip configuration and aeration-slot arrangement, and pointed out that the degree of negative pressure downstream of the valve was related to valve opening, the upstream–downstream head difference, and downstream-submergence depth, with the minimum pressure generally occurring at relative valve openings of 0.3–0.5.

Considerable efforts have been devoted to transitions between free-surface and pressurized flows, as well as air entrainment in tailrace tunnels and urban drainage systems. Liu et al. [12] employed the VOF method together with a dynamic mesh technique to simulate an air–water two-phase flow during gate opening in a bottom spillway and predicted the amount of air entrainment in the gate shaft and downstream conduit induced by plunging flow. Their results showed that the initial conduit water level and the gate-opening procedure strongly influenced air entrainment in the gate shaft. Lu et al. [13] used a three-dimensional CFD approach to simulate water–air coupling in an undulating pipeline, revealing the dynamic characteristics of compression, expansion, and deformation of entrapped air pockets. Shi et al. [14] applied the VOF method to analyze the three-dimensional flow evolution during free-surface–pressurized flow transitions in a tailrace system, and showed that the direction of vent airflow was closely related to the rise and fall of the downstream water level, although no distinct periodicity was observed. Li et al. [15] developed a numerical model for free-surface–pressurized flow by combining the realizable k–ε model with the VOF method and simulated the pressure response of a tailrace system subjected to downstream river-level variations at different rates. Their results indicated that downstream water-level fluctuations could induce regular pressure oscillations accompanied by numerous vortex structures. Zhou et al. [16] employed a VOF model coupled with a compressible water–air two-phase formulation to investigate the transient effects of load variations on discharge, water level, and pressure response in a tailrace tunnel under open-channel flow, pressurized flow, and free-surface–pressurized transition conditions. Their results showed that the outlet discharge of the tailrace tunnel exhibited damped oscillations in response to load variations, while the free-surface–pressurized transition was generally accompanied by pronounced pressure fluctuations. Hu et al. [17] conducted physical model experiments to examine the transition between open-channel and pressurized flows and the associated air–water interactions in urban drainage pipelines, focusing on pressure response, air-pocket migration, and wave propagation under different combinations of inflow discharge and downstream submergence. Chen et al. [18] divided the pipe-filling process into four stages based on physical model tests and established an empirical relationship between the maximum pressure and the air-release rate. Zhang et al. [19] investigated flow-regime evolution, air-pocket transport, and the responses of hydraulic parameters during the transition from free-surface flow to pressurized flow in an open-channel tunnel conveyance system.

These studies have advanced the understanding of free-surface–pressurized flow transitions, air–water interactions, and transient air-entrainment processes in conduits and provide useful references for interpreting unsteady hydraulic behavior in valve sections and shaft aeration. Nevertheless, most existing studies have focused on air–water interactions induced by free-surface pipe flow, rapid filling, or plunging flow, whereas post-valve aeration in the downstream shaft of a navigation-lock filling-and-emptying valve under extreme downstream water-level conditions remains insufficiently investigated.

In this study, a three-dimensional CFD model was established to investigate the post-valve aeration process and its evolution in the downstream shaft of a navigation-lock filling-and-emptying valve under different combinations of inlet velocity and downstream-submergence depth. In contrast to most studies on air entrainment in conveyance systems, where air is initially present within the conduit, the air–water flow considered here is generated by air intake through the downstream shaft and subsequent transport by the water flow. The main contributions of this work are as follows: first, the air-entrainment process and characteristic flow regimes during the later stage are analyzed under different inlet velocities and downstream-submergence depths; second, the entrapped-air-pocket volume, pressure distribution, shear-layer instability characteristics, and evolution of vortical structures are quantitatively evaluated; and third, the pressure responses at representative monitoring points in the shaft and post-valve culvert system are examined to clarify the relationship between pressure variations and entrapped-air-pocket evolution. These results provide a reference for identifying aeration states and assessing entrapped-air-pocket risks in downstream shafts of navigation-lock water conveyance systems.

## 2. Mathematical Models

This study focuses on the aeration process and entrapped-air-pocket evolution in the downstream shaft of a filling-and-emptying system. The flow-field structure, air–water interface evolution, entrapped-air-pocket volume, and pressure responses in the shaft and near-valve regions were analyzed. The RNG k–ε turbulence model was adopted to describe the turbulent flow because of its established robustness in strongly sheared and recirculating engineering flows [20]. The volume-of-fluid (VOF) method was employed to capture the transient air–water interface. The present RANS–VOF framework is intended to characterize the macroscopic unsteady flow, large-scale vortical structures, and air–water interface evolution; small-scale turbulent eddies and bubble-scale breakup and coalescence are beyond the resolution of the present model and would require higher-fidelity approaches such as LES/DES or interface-resolved multiphase modeling. The governing equations are given below.

Continuity equation

(1) $$\frac{\partial \rho }{\partial t}+\frac{\partial \rho u_{i}}{\partial x_{i}}=0$$

Momentum equation

(2) $$\frac{\partial \rho u_{i}}{\partial t}+\frac{\partial }{\partial x_{j}}(\rho u_{i}u_{j})=-\frac{\partial p}{\partial x_{i}}+\frac{\partial }{\partial x_{j}}\left[\left(\mu +\mu_{t})(\frac{\partial u_{i}}{\partial x_{j}}+\frac{\partial u_{j}}{\partial x_{i}}\right)\right]+\rho g_{i}$$

*k* equation

(3) $$\frac{\partial (\rho k)}{\partial t}+\frac{\partial (\rho u_{i}k)}{\partial x_{i}}=\frac{\partial }{\partial x_{i}}\left[(\mu +\frac{\mu_{t}}{\sigma_{k}})\frac{\partial k}{\partial x_{i}}\right]+G_{k}-\rho \varepsilon$$

ε equation

(4) $$\frac{\partial (\rho \varepsilon )}{\partial t}+\frac{\partial (\rho u_{i}\varepsilon )}{\partial x_{i}}=\frac{\partial }{\partial x_{i}}\left[(\mu +\frac{\mu_{t}}{\sigma_{\varepsilon }})\frac{\partial \varepsilon }{\partial x_{i}}\right]+C_{1\varepsilon }^{*}\rho \frac{\varepsilon }{k}G_{k}-C_{2\varepsilon }\rho \frac{\varepsilon^{2}}{k}$$

where *ρ* and *μ* denote the mixture density and dynamic viscosity, respectively, and are determined from the local phase volume fractions. They are given by $\rho =\alpha_{w}\rho_{w}+\left(1-\alpha_{w}\right)\rho_{a}$ and $\mu =\alpha_{w}\mu_{w}+\left(1-\alpha_{w}\right)\mu_{a}$, respectively, where $\alpha_{w}$ = water volume fraction and *α_a_* = 1 − *α_w_* is the air volume fraction; *ρ_w_* and *ρ_a_* are the densities of water and air, respectively; $\mu_{w}$ and $\mu_{a}$ are viscosities of water and air, respectively; *p* = pressure; *t* = time; *k* = turbulent kinetic energy; *ε* = dissipation rate of turbulent kinetic energy; and *μ_t_* = turbulent viscous coefficient, which can be derived from:

(5) $$\mu_{t}=\rho C_{\mu }\frac{k^{2}}{\varepsilon }$$

$G_{k}$ is the turbulent kinetic energy produced by the mean velocity gradients.

(6) $$G_{k}=\mu_{t}\left(\frac{\partial u_{i}}{\partial x_{j}}+\frac{\partial u_{j}}{\partial x_{i}}\right)\frac{\partial u_{i}}{\partial x_{j}}$$

(7) $$C_{1\varepsilon }^{*}=1.42-\frac{\eta }{1+\beta \eta^{3}}\left(1-\frac{\eta }{\eta_{0}}\right)$$

(8) $$\eta =\frac{Sk}{\varepsilon }$$

(9) $$S=\sqrt{2{\bar{S}}_{ij}{\bar{S}}_{ij}}$$

(10) $${\bar{S}}_{ij}=\frac{1}{2}\left(\frac{\partial u_{i}}{\partial x_{j}}+\frac{\partial u_{j}}{\partial x_{i}}\right)$$

The values of the parameters are shown in Table 1.

The VOF method is a classical numerical approach for resolving free-surface flows and gas–liquid interfacial dynamics. It has been widely validated and applied to problems involving free-surface fluctuations, air–water mixing, and large-scale interfacial deformation [21,22,23]. In this method, a phase volume-fraction function is introduced on a fixed Eulerian grid to describe the volumetric occupancy of each phase within a computational cell, thereby tracking the spatiotemporal evolution of the gas–liquid interface [24]. Within the VOF framework, each computational cell is occupied by water and air, and the sum of their volume fractions is equal to unity. Let *α_w_* denote the water volume fraction. Accordingly, $\alpha_{w}$ = 0 indicates that the control volume is fully occupied by air, *α_w_* = 1 indicates that it is fully occupied by water, and 0 < $\alpha_{w}$ < 1 denotes a cell containing both water and air, corresponding to an air–water mixed region or an interface-capturing region.

The spatiotemporal evolution of the air–water interface is obtained by solving the interface-tracking transport equation:

(11) $$\frac{\partial \alpha_{w}}{\partial t}+u_{i}\frac{\partial \alpha_{w}}{\partial x_{i}}=0$$

To further quantify the formation and evolution of the entrapped air pocket downstream of the valve, the gas-phase field was filtered based on gas-dominated regions. Specifically, only computational cells with an air volume fraction of $\alpha_{a}$ > $\alpha_{\mathrm{th}}$ = 0.8 within the fixed post-valve culvert domain $D_{c}$ were included in the volume integration, thereby reducing the influence of external free air and dilute aerated regions on the statistical results. Accordingly, the unit-width entrapped-air-pocket volume, which characterizes the gas-dominated air-pocket region in the post-valve culvert, is defined as:

(12) $$V_{b}=\frac{1}{W}\int_{D_{c}}I(\alpha_{a}>\alpha_{\mathrm{th}})\alpha_{a}dV$$

where $V_{b}$ denotes the unit-width entrapped-air-pocket volume, W is the width of the reduced-width computational model, $D_{c}$ denotes the fixed sampling domain inside the post-valve culvert, $\alpha_{a}$ is the air volume fraction, and $\alpha_{\mathrm{th}}$ is the air-pocket identification threshold, which was set to 0.8 in this study. *I*(·) is an indicator function that equals 1 when the specified condition is satisfied and 0 otherwise, and *dV* denotes the differential volume element [25]. Since the computational model width is *W* = 0.5 m, all reported air-pocket volume indices are normalized by this width and expressed as unit-width values with a unit of m^2^. Air was treated as a constant-density phase; therefore, *V_b_* was mainly used to characterize air-pocket retention and evolution rather than thermodynamic compression.

## 3. Simulation Setup

### 3.1. Computational Domain and Boundary Conditions

The flat-bottomed culvert with a gradually expanding roof is widely used in navigation-lock water conveyance systems. After valve opening, this type of culvert tends to generate high-speed jets, recirculation separation, and local low-pressure regions, resulting in a complex and strongly unsteady post-valve flow. In this study, a three-dimensional geometric model of the reverse tainter valve section and the downstream shaft was established. The downstream shaft in this study refers to a vertical structure connected to the post-valve culvert, which may correspond to a downstream maintenance shaft or an air vent in different engineering applications. The computational domain consists of the inlet section, reverse tainter valve and valve well, downstream shaft, and outlet section.

**Figure 1:**
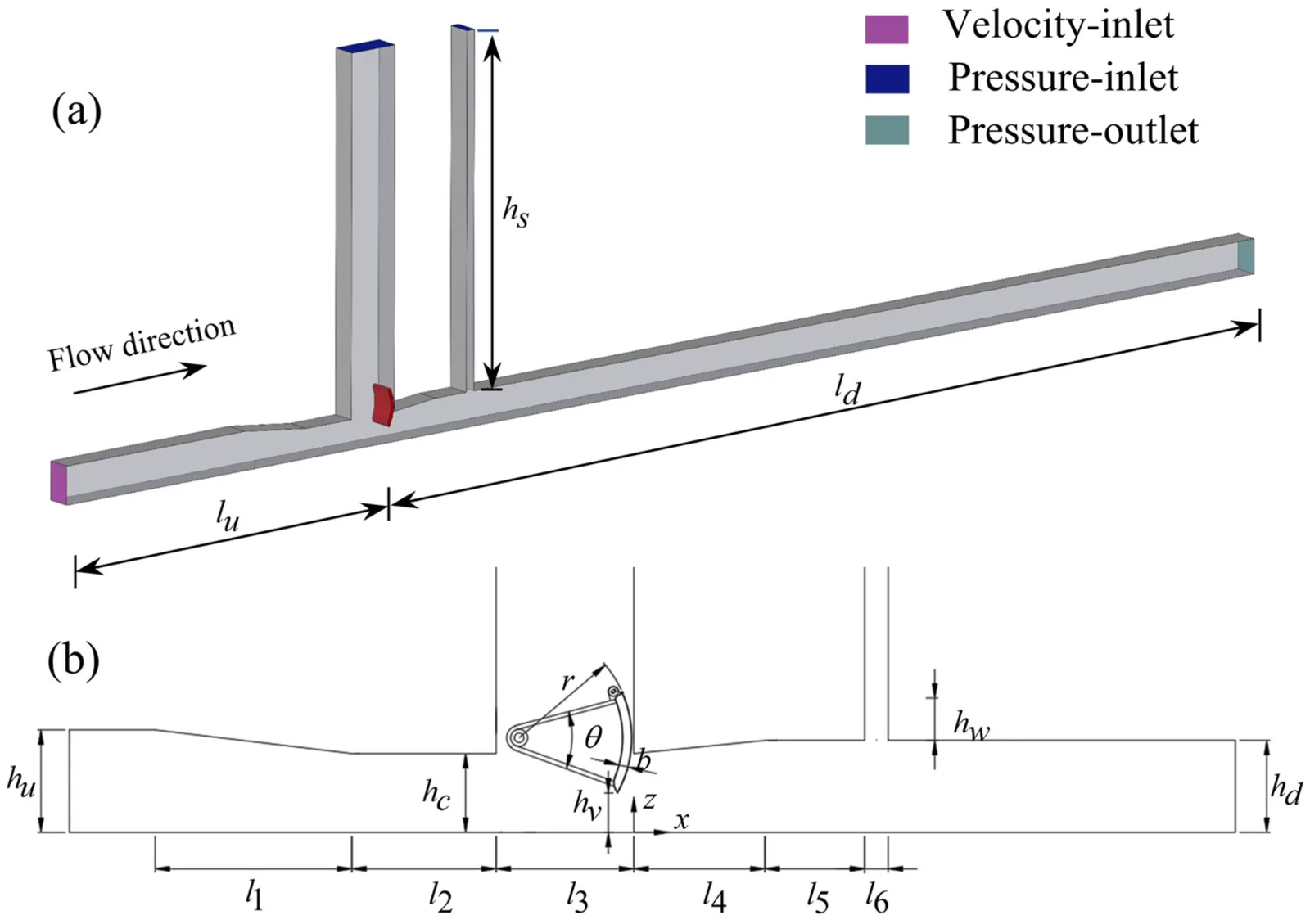
Schematic diagram of the computational domain: (**a**) computational domain and boundary-condition setup; (**b**) local geometric dimensions of the valve section. The red component represents the reverse tainter valve.

Figure 1 shows the schematic layout of the valve section and downstream shaft. The coordinate system was established according to the right-hand rule, with the origin located at the intersection between the downstream wall of the valve well and the culvert bottom (Figure 1b). The main flow direction in the culvert is defined as the *x*-axis, the transverse direction as the *y*-axis, and the vertical upward direction as the *z*-axis. In terms of geometric dimensions, the horizontal lengths of the upstream and downstream culverts are $l_{u}$ = 70 m and $l_{d}$ = 200 m, and the vertical height from the downstream shaft to the culvert roof is $h_{s}$ = 63 m. The inlet-section, valve-section, and outlet-section heights are $h_{u}$= 7.8 m, $h_{c}$ = 6.0 m, and $h_{d}$ = 7.0 m, while $h_{w}$ denotes the downstream-submergence depth. The local dimensions of the valve section are $l_{1}$ = 15.0 m, $l_{2}$ = 11.0 m, $l_{3}$ = 10.5 m, $l_{4}$ = 11.0 m, and $l_{5}$ = 10.5 m, and the horizontal length of the valve well is $l_{6}$ = 1.8 m. The reverse tainter valve has a radius of *r* = 8.6 m, a central angle of θ = 34.6°, and a valve-body thickness of *b* = 0.7 m.

The prototype valve section is 5.0 m wide, whereas a 0.5 m reduced-width 3D model with lateral symmetry boundaries was adopted to reduce computational cost. Previous studies support this sectional modeling approach: Ma et al. [7] reported similar flow fields from two- and three-dimensional simulations of the Three Gorges Lock filling culvert, while Battiston et al. [26] showed that a two-dimensional RANS model reasonably reproduced experimental post-valve pressure and velocity characteristics. The present model is therefore used to capture the dominant streamwise–vertical flow and air-pocket evolution for comparative analysis among different hydraulic conditions.

As shown in Figure 1a, a velocity-inlet boundary condition was imposed at the upstream boundary. The inlet-velocity range was estimated from the measured chamber water-level variation under different valve openings using Equation (13). A hydrostatic-pressure boundary condition was prescribed at the downstream boundary, and the corresponding downstream-submergence depth *h_w_* was specified through a user-defined function. The valve well and downstream shaft were both specified as atmospheric pressure-inlet boundaries with a gauge pressure of 0 Pa, with air specified as the backflow phase. This pressure condition was applied only at their top openings, while the internal pressure fields evolved under gravity and local flow dynamics. The two lateral side boundaries of the computational model were specified as symmetry boundaries. All remaining solid boundaries were treated as no-slip stationary walls. The initial water-phase distribution was set according to the prescribed downstream-submergence depth, with the region below the downstream still-water level initialized as water.

The three-dimensional CFD simulations were performed using ANSYS Fluent 2022 R1. The VOF equations were solved using an implicit formulation with sharp interface modeling. The gravitational acceleration was set to 9.81 m/s^2^ in the negative *z*-direction. The convective terms in the governing equations were discretized using a second-order upwind scheme, and the pressure–velocity coupling was handled using the PISO algorithm. According to the Courant-number constraint, the time step was set to Δ*t* = 0.01 s to balance numerical stability and computational efficiency. The convergence criterion was specified such that the residuals of all governing equations were below 10^−5^.

(13) $$v_{i}=\frac{S\Delta h}{2S_{i}\Delta t}$$

For the synchronous opening of two lock valves, *S* is the lock chamber area, *S_i_* is the culvert cross-sectional area, and Δ*h* is the variation in chamber water level within the time interval Δ*t*.

Based on the estimated inlet-velocity range, four representative constant inlet velocities (*v_i_* = 3–6 m/s) were selected to cover the main operating range of the post-valve flow. The valve opening was fixed at *n* = 0.5, corresponding to the opening condition under which a pronounced post-valve low-pressure region was likely to occur. Four downstream-submergence depths (*h_w_* = 0–8 m) were considered to represent different degrees of downstream water-level confinement. The resulting computational cases are summarized in Table 2. The 16 cases were used to evaluate the overall effects of inlet velocity and downstream submergence on shaft aeration and entrapped-air-pocket evolution. On this basis, four cases at *v_i_* = 5 m/s with different downstream-submergence depths were selected for detailed flow-field analysis to compare the vortex structures, shear-layer characteristics, and pressure responses associated with different aeration regimes under a fixed inlet condition.

### 3.2. Mesh Generation and Independence Verification

The computational domain was discretized using structured meshes, with local refinement applied to the key flow regions between the valve well and the downstream shaft. The refined regions mainly included the high-speed post-valve jet region, shear layer, recirculation region, and the area extending approximately 15 m downstream of the downstream shaft.

**Figure 2:**
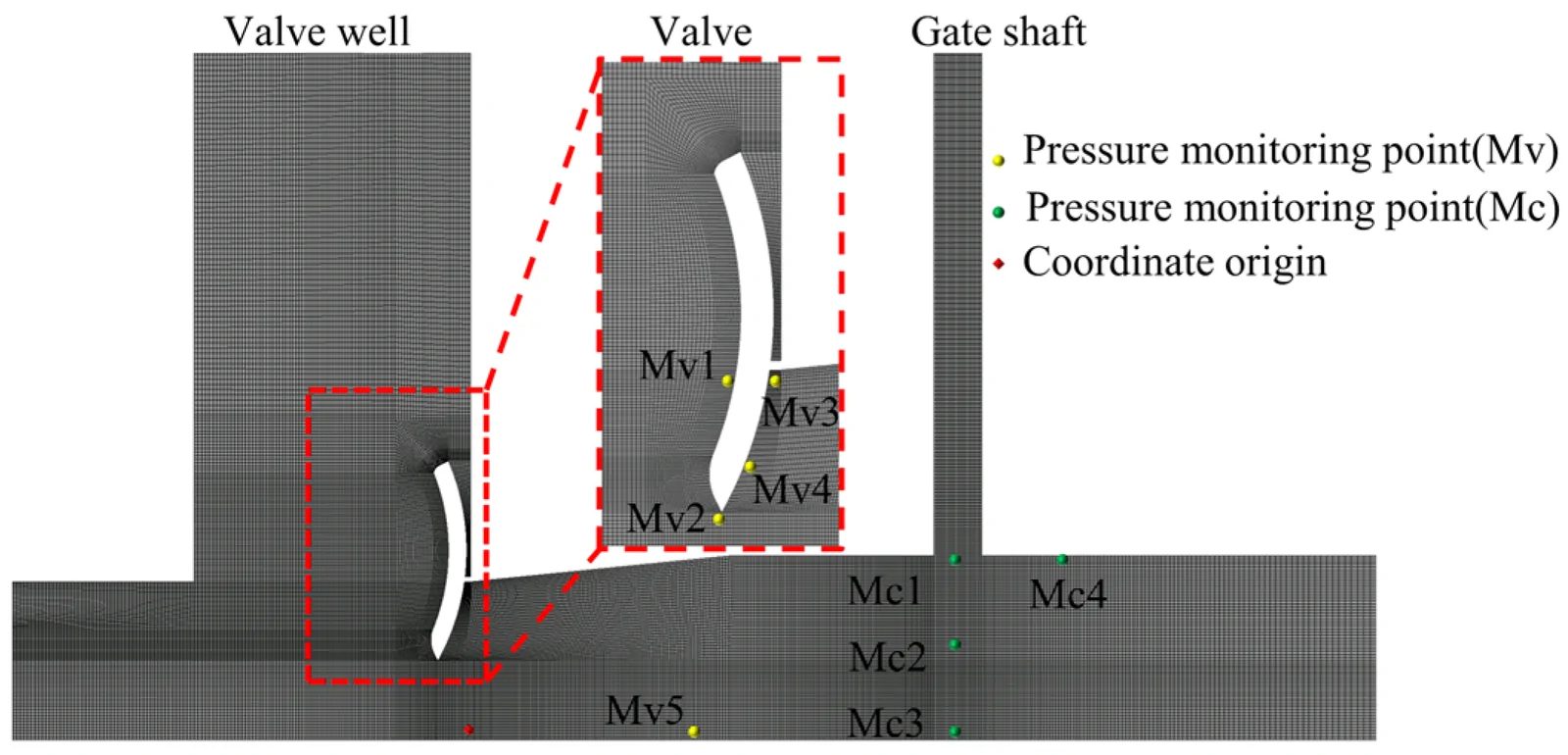
Mesh discretization and arrangement of pressure monitoring points.

To analyze the pressure response near the valve and the shaft, monitoring points Mv and Mc were arranged around the valve and the downstream shaft, respectively. Specifically, Mv1 was used to monitor the pressure response on the upstream face of the valve, while Mv2–Mv4 were used to monitor the pressure response on the downstream face. Mv5 was located at *x* = 9 m from the coordinate origin to monitor pressure variations in the post-valve jet core region. The Mc monitoring points were arranged to capture the pressure response at different elevations during the post-valve aeration process in the downstream shaft. Mc1–Mc3 were located in the upper, middle, and bottom parts of the shaft, respectively, whereas Mc4 was positioned 6 m downstream of Mc1 to monitor the pressure response on the downstream side of the valve well. The detailed arrangement is shown in Figure 2.

**Figure 3:**
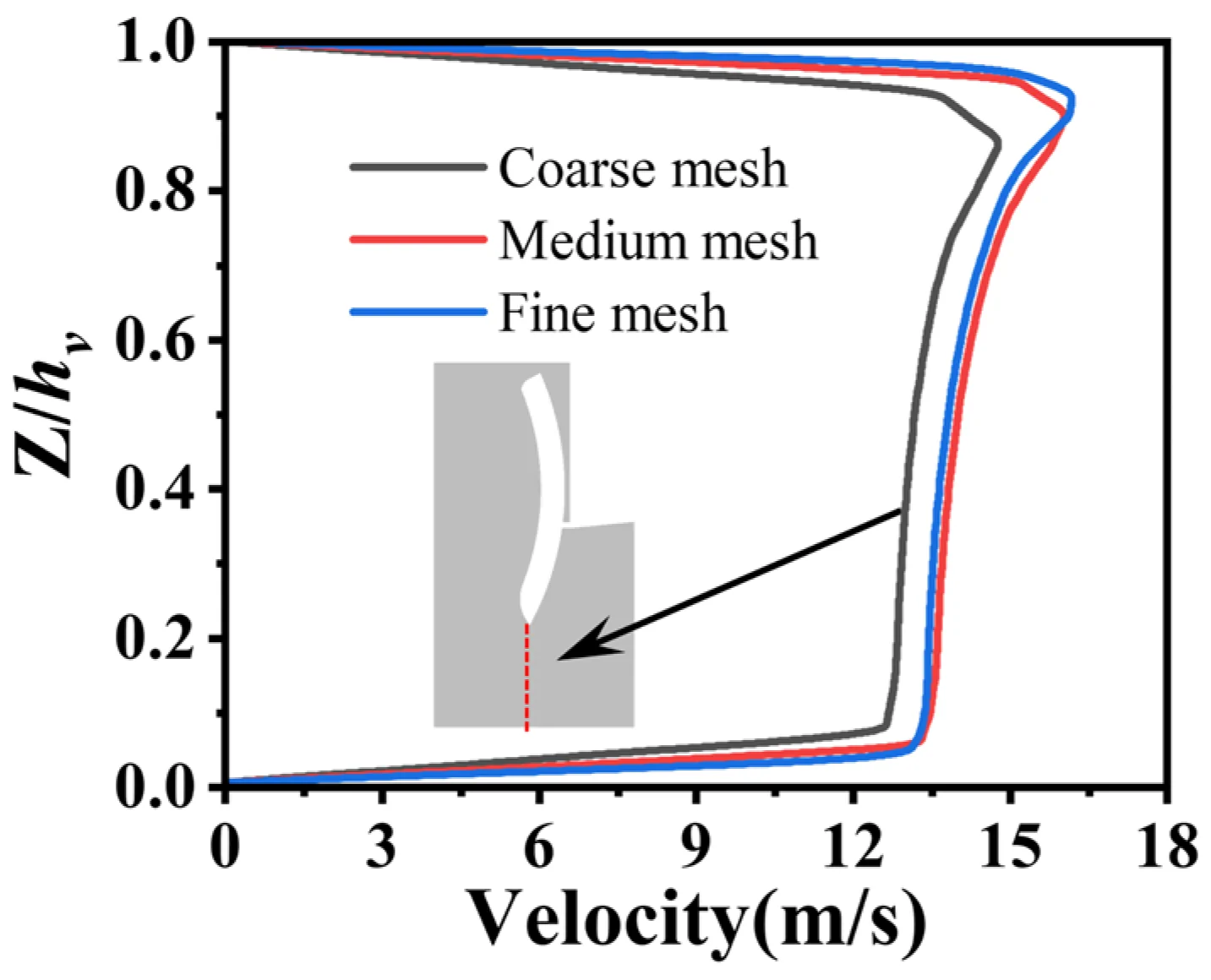
Vertical profiles of the streamwise velocity at the valve-opening section obtained using different mesh resolutions.

To verify grid independence, a representative case with an inlet velocity of *v_i_* = 5 m/s and a downstream-submergence depth of *h_w_* = 0 m was selected for analysis. Three mesh systems were generated, with 0.512, 1.116, and 1.988 million cells, respectively. The vertical profile of the streamwise velocity at the valve-opening section (*x* = −1.2 m) at *t* = 200 s was extracted, as shown in Figure 3 and Table 3**.** As the mesh was progressively refined, the velocity distributions obtained using the three meshes became generally consistent; $\bar{v}$ increased from 12.42 to 13.59 m/s, while $v_{\max}$ increased from 14.87 to 16.29 m/s. Compared with the coarse mesh, the medium mesh was able to capture the main velocity-distribution characteristics at the valve-opening section. Further refinement from the medium to the fine mesh resulted in only minor changes. The relative differences in $v_{\max}$ between the coarse and fine meshes and between the medium and fine meshes were 8.72% and 0.80%, respectively.

**Figure 4:**
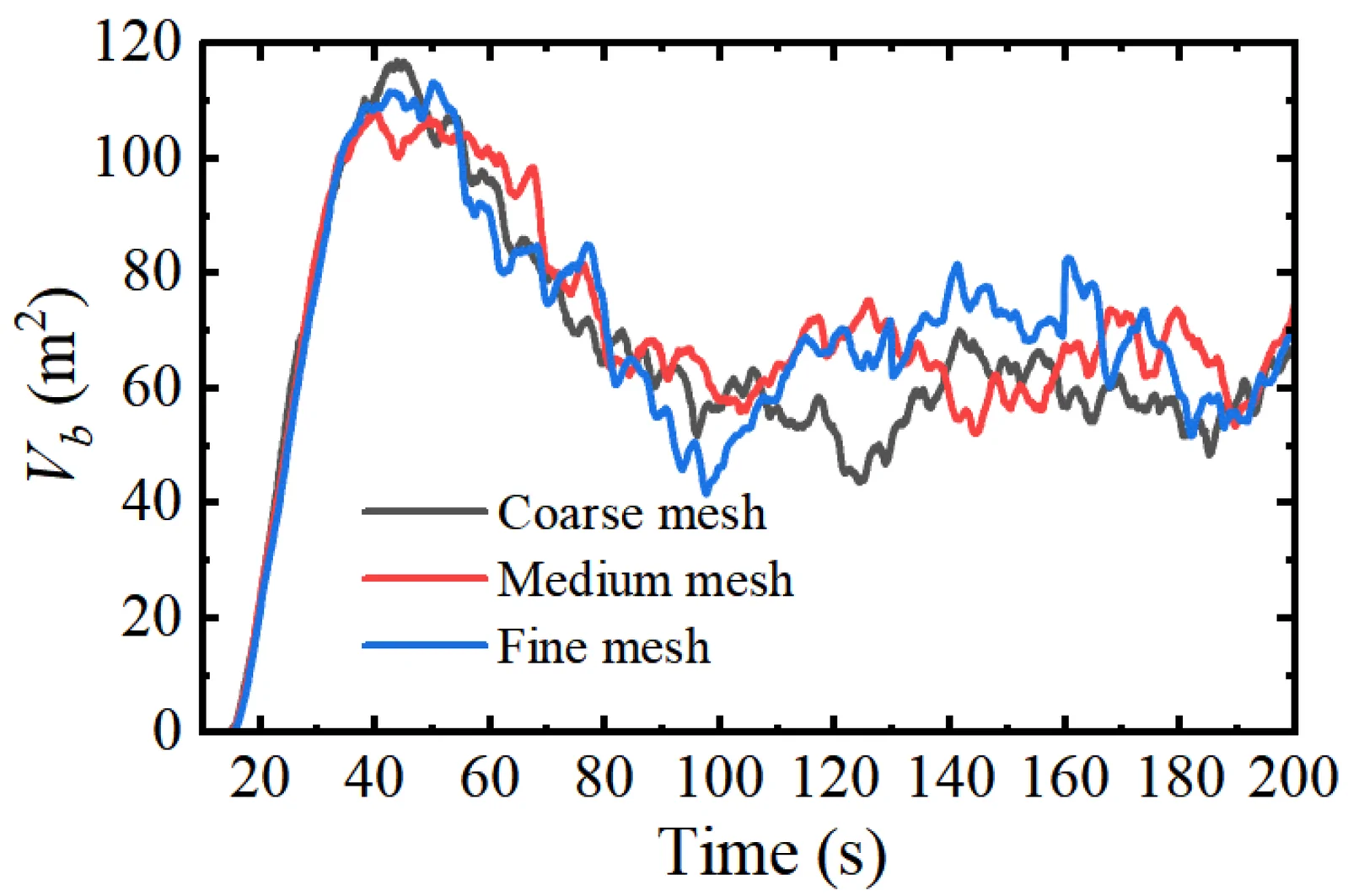
Temporal evolution of unit-width entrapped-air-pocket volume under different mesh resolutions.

Mesh sensitivity was further evaluated using the late-stage mean unit-width entrapped-air-pocket volume *V_b_*_,s_, averaged over *t* = 100–200 s. As shown in Figure 4, the values of *V_b_*_,s_ obtained using the coarse, medium, and fine meshes were 58.06, 66.50, and 69.27 m^2^, respectively. Relative to the fine-mesh result, the difference in *V_b_*_,s_ decreased from 16.18% for the coarse mesh to 4.00% for the medium mesh. Meanwhile, the relative difference in *v*_max_ between the medium and fine meshes was only 0.80%, and their velocity profiles at the valve-opening section were also in close agreement. These results indicate that further refinement from the medium to the fine mesh produces only limited changes in the principal quantities considered in this study. Therefore, considering both numerical accuracy and computational cost, the medium mesh was adopted for the subsequent simulations.

### 3.3. Numerical Model Validation

To verify the reliability of the numerical model, the experimental results for a reverse tainter valve reported by Battiston et al. [26] were selected for comparison. The detailed geometric configuration and boundary conditions used to reconstruct the validation model were obtained from the doctoral thesis of Battiston [27]. The validation was conducted under the 50% valve-opening condition with a discharge of 180 L/s, and the longitudinal pressure distribution along the culvert invert downstream of the valve was used as the validation metric.

**Figure 5:**
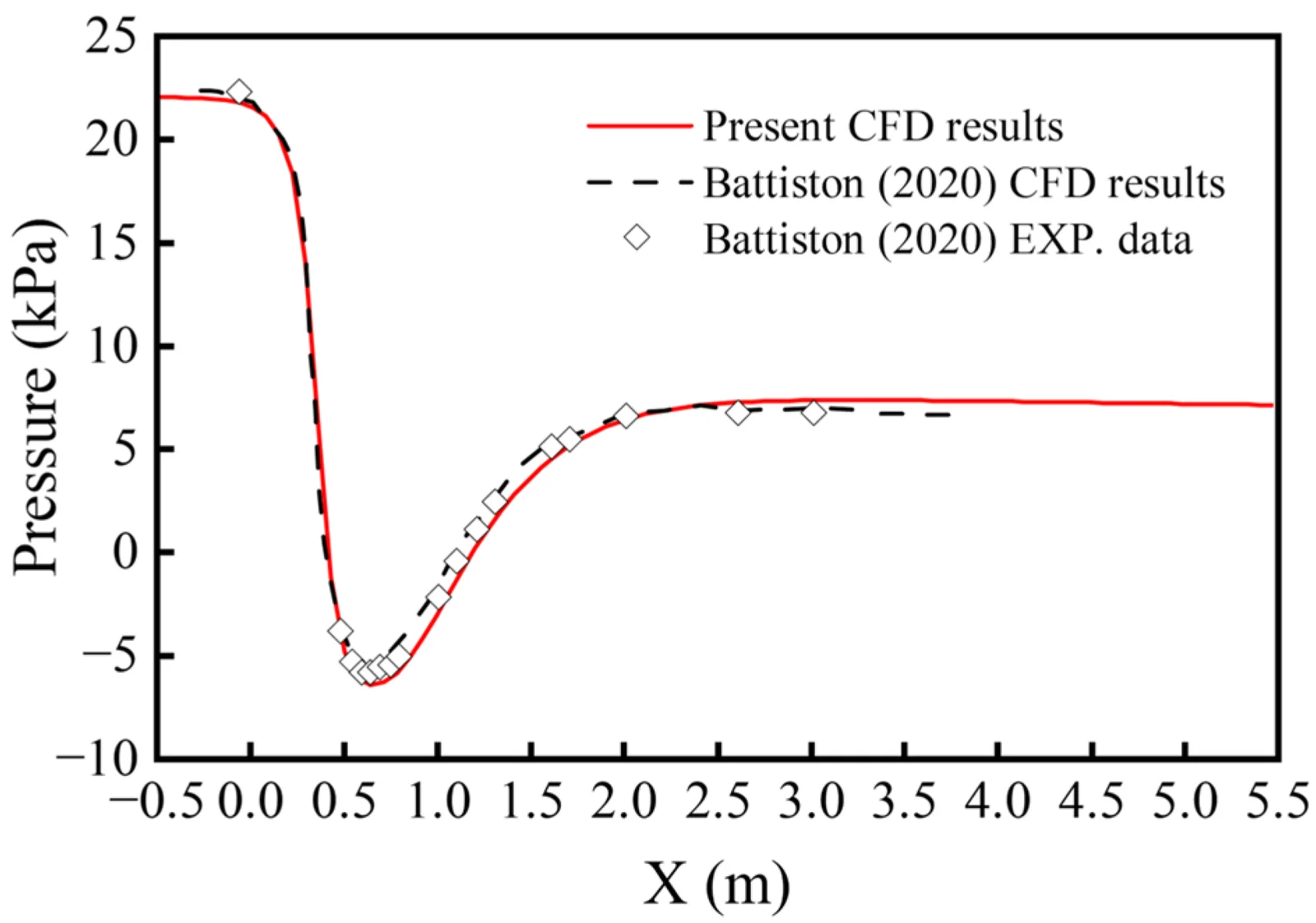
Comparison of pressure distributions along the culvert invert at a relative valve opening of *n* = 0.5 [26].

As shown in Figure 5, the present numerical results are generally consistent with the numerical results reported by Battiston et al. [26] and agree well with the experimental data. The model reasonably reproduces the rapid pressure drop downstream of the valve, the formation of the low-pressure region, and the subsequent pressure recovery along the culvert. The location of the minimum pressure values and the overall pressure-recovery trend are also consistent with the experimental observations. Quantitatively, the comparison yielded a mean absolute error (MAE) of 0.56 kPa, a root-mean-square error (RMSE) of 0.61 kPa, and a normalized root-mean-square error (NRMSE) of 2.15%, where the NRMSE was normalized by the range of the experimental pressure data. In addition, the difference between the calculated and measured minimum pressures was approximately 0.62 kPa. These results indicate that the model can reasonably predict the low-pressure region and pressure-recovery process downstream of the valve.

In engineering design and operation, sustained air intake through the downstream maintenance shaft is generally avoided; therefore, prototype-scale observations of shaft aeration and entrapped-air-pocket evolution are difficult to obtain. Accordingly, the two-dimensional validation was used primarily to assess the post-valve pressure response and the prediction of the low-pressure region under pressurized flow conditions, rather than the full three-dimensional air-pocket dynamics. The validation results show that the established numerical model can reasonably reproduce the main characteristics of the pressure distribution downstream of the valve. Since the development of sub-atmospheric pressure provides the hydraulic driving condition for downstream-shaft aeration, the reproduced negative-pressure characteristics also provide indirect support for the prediction of shaft-aeration tendency. Previous VOF-based studies have also demonstrated the capability of this approach to reproduce air–water interface evolution and entrapped-air-pocket dynamics in related conduit flows [12,25]. On this basis, the model is considered suitable for comparative analysis of post-valve flow behavior, downstream-shaft-aeration tendency, and entrapped-air-pocket evolution under different hydraulic conditions.

## 4. Results and Discussion

### 4.1. Flow-Regime Transition and Air-Entrainment Patterns in the Downstream Shaft

This section first examines the complete aeration process in the post-valve shaft and classifies the late-time flow regimes under different hydraulic conditions. On this basis, the entrapped-air volume index proposed above is used to evaluate post-valve shaft aeration under different inlet velocities and downstream-submergence depths, and the transition interval from transient to sustained shaft aeration is identified under the investigated conditions.

#### 4.1.1. Air Entrainment and Flow-Regime Evolution

**Figure 6:**
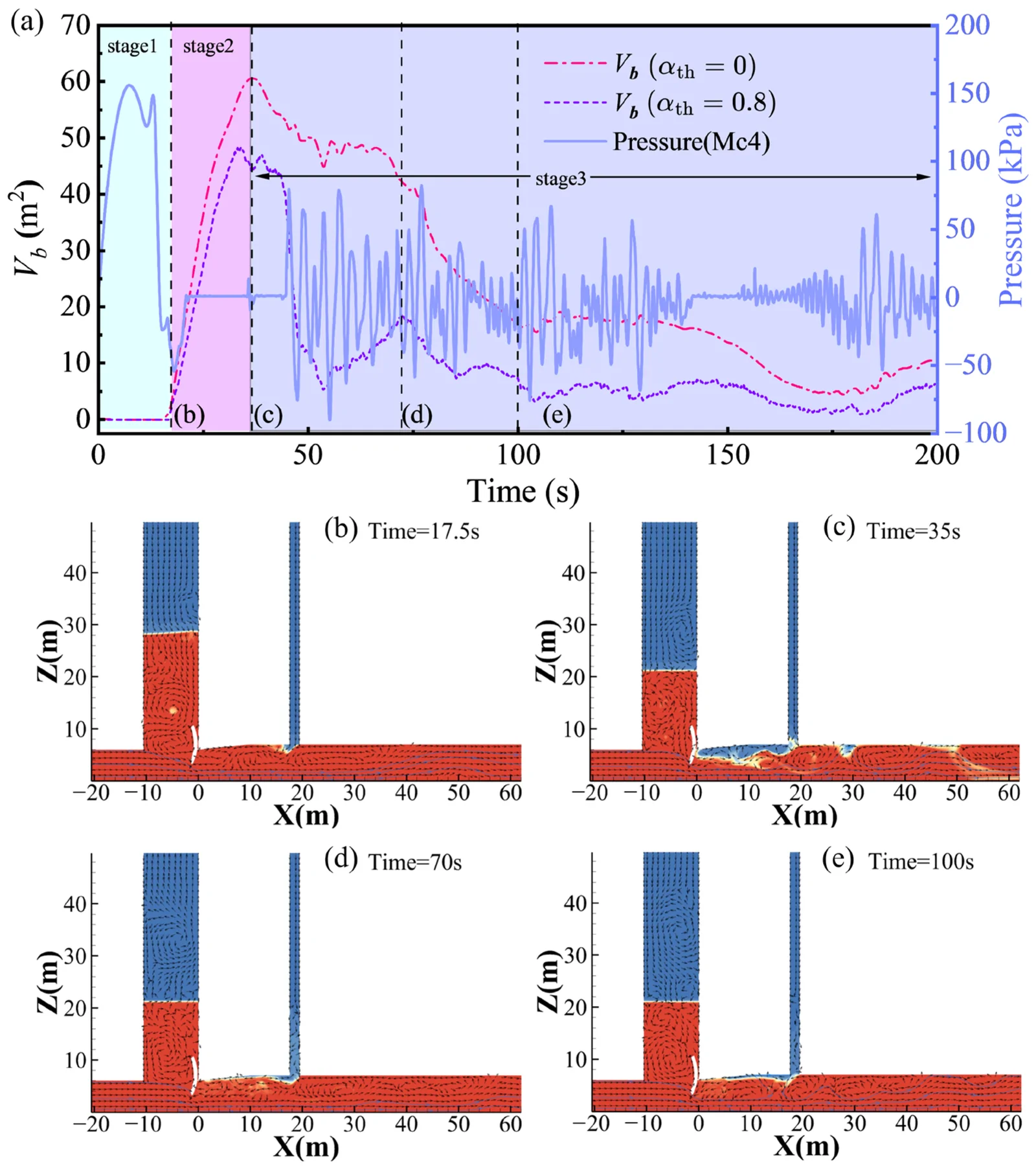
Temporal evolution of downstream-shaft aeration at *v_i_* = 5 m/s and *h_w_* = 2 m: (**a**) unit-width gas volumes identified using *α*_th_ = 0 and *α*_th_ = 0.8, together with the static pressure at Mc4; (**b**–**e**) instantaneous air–water phase distributions at *t* = 17.5, 35, 70, and 100 s, respectively. In panels (**b**–**e**), red and blue denote water and air, respectively.

Figure 6 presents the temporal variations in the entrapped-air-pocket volume downstream of the valve and the pressure at monitoring point Mc4 under the representative operating condition of *v_i_* = 5 m/s and *h_w_* = 2 m. Both the entrapped-air-pocket volume and the pressure response exhibit pronounced stage-dependent characteristics. Based on the coupled evolution of the pressure response and gas-phase distribution, the downstream-shaft-aeration process can be divided into three stages: jet establishment, coherent air-pocket formation, and air-pocket breakup and reorganization. After approximately *t* = 100 s, the instantaneous flow remains strongly fluctuating, but its statistical characteristics no longer exhibit a persistent increasing or decreasing trend.

The first stage is jet establishment (*t* = 0–17 s). During this stage, the pressure in the downstream shaft rises rapidly and then drops sharply, whereas the entrapped-air-pocket volumes identified using both criteria remain close to zero. This indicates that the flow is mainly governed by the formation of the high-speed jet downstream of the valve and the associated local pressure build-up. At this stage, no persistent entrapped air pocket has yet formed in the post-valve region.

The second stage corresponds to air-pocket formation (*t* = 17–40 s). During this stage, the pressure in the downstream shaft exhibits an early pressure trough of approximately −53 kPa. Meanwhile, the entrapped-air-pocket volumes identified using both criteria increase rapidly and reach their peak values at approximately *t* = 37 s. This indicates that, as the low-pressure region and shear layer downstream of the valve progressively develop, sustained air intake occurs through the shaft, leading to the rapid formation and downstream expansion of a coherent entrapped air pocket. The close temporal correspondence between the pressure trough and the rapid increase in entrapped-air-pocket volume suggests that the dominant flow mechanism shifts from jet establishment to sustained air entrainment during this stage.

The third stage corresponds to unsteady air-pocket breakup and reorganization (*t* > 40 s). Thereafter, the pressure in the downstream shaft enters a strongly oscillatory state. During this stage, the air volume identified using the high-threshold criterion with *α*_th_ = 0.8 decreases markedly from its peak value, whereas that identified using the low-threshold criterion with *α*_th_ = 0 remains at a relatively high level. This indicates that the coherent entrapped air pocket formed in the preceding stage does not disappear as a whole. Instead, under the combined effects of shear action, recirculation development, and interfacial disturbances in the shaft, it breaks up and reorganizes, gradually transforming into numerous dispersed small air pockets and low-air-volume-fraction regions. Therefore, the low-threshold criterion with *α*_th_ = 0 is more suitable for describing the overall air-affected region, whereas the high-threshold criterion with *α*_th_ =0.8 better characterizes the coherent entrapped air pocket. In the following analysis, the air volume identified using the high-threshold criterion with *α*_th_ = 0.8 is used to represent the entrapped-air-pocket volume. The robustness of the subsequent flow-regime classification to the selected threshold is further examined in Section 4.1.3. During the early part of this stage, the pressure and air-pocket volume undergo pronounced adjustments. After approximately *t* = 100 s, their mean levels and overall fluctuation ranges become approximately stationary, although substantial instantaneous oscillations persist.

To further elucidate the instantaneous evolution of downstream-shaft aeration at different stages, several representative instants are marked by dashed lines in Figure 6a, and the corresponding air–water phase distributions are presented in Figure 6b–e. In these contours, red denotes the water phase, blue denotes the air phase, and the black arrows indicate the flow direction.

Figure 6b corresponds to the transition from Stage 1 to Stage 2, at approximately *t* = 17.5 s. At this instant, the air–water interface in the downstream shaft (*x* = 18.5 m) begins to show a localized downward depression. Air in the shaft is entrained by the low-pressure region of the main flow, but the air phase remains mainly confined near the culvert roof and has not yet developed into a distinct downstream air-transport structure. This indicates that a stable pressure-difference-driven exchange between the main flow and the downstream shaft has not yet been established.

Figure 6c corresponds to the stage when downstream-shaft aeration approaches its peak, at approximately *t* = 35 s. At this moment, air intake through the downstream shaft is markedly enhanced, and the entrapped air pocket beneath the culvert roof reaches its maximum extent, accompanied by pronounced air–water interface instability. Continuous air entrainment is observed near *x* = 18.5 m, while a bubbly flow develops along the culvert roof and propagates downstream over *x* = 20–50 m. The entrapped air first expands upstream along the culvert crown toward the valve region; after the roof air pocket extends back to the near-valve region, part of the entrapped air is carried downstream by the main flow. Meanwhile, new air-entrainment events continue to occur near the shaft opening.

Figure 6d corresponds to a local re-entrainment peak during Stage 3, at approximately *t* = 70 s. Before this moment, air intake through the downstream shaft has already become substantial, and the negative pressure in the culvert is partly relieved, causing part of the air to be released through the shaft. However, the pressure near the shaft continues to fluctuate strongly between positive and negative values, allowing weak local air re-entrainment to occur again. This behavior indicates that, during Stage 3, the entrapped air pocket undergoes increasingly intense oscillation, accompanied by local breakup and reorganization, which results in a more irregular flow structure.

Figure 6e shows the flow field at *t* = 100 s, corresponding to the beginning of the statistical averaging interval. Although the pressure in the downstream shaft still alternates between positive and negative values, a moderate amount of air is continuously entrained to compensate for the local negative pressure. The flow near the shaft opening therefore becomes more stable, the air–water interface gradually smooths out, and the air-pocket distribution in the main-flow region becomes more organized. At this time, the pressure and air-pocket volume continue to fluctuate, but their mean levels and overall fluctuation ranges no longer exhibit a persistent temporal trend. Therefore, *t* = 100–200 s was selected as the statistical averaging interval rather than being defined as a steady-flow stage.

It is worth noting that distinct air-transport pathways are observed in the shear-layer region at all representative instants. This behavior can be attributed to the high main-flow velocity and the associated strong pressure drop. With continuous air supply from the downstream shaft, the shear layer becomes increasingly unstable, thereby promoting air entrainment, air-pocket breakup, and downstream air transport.

#### 4.1.2. Evolution of Downstream Flow Regimes

**Figure 7:**
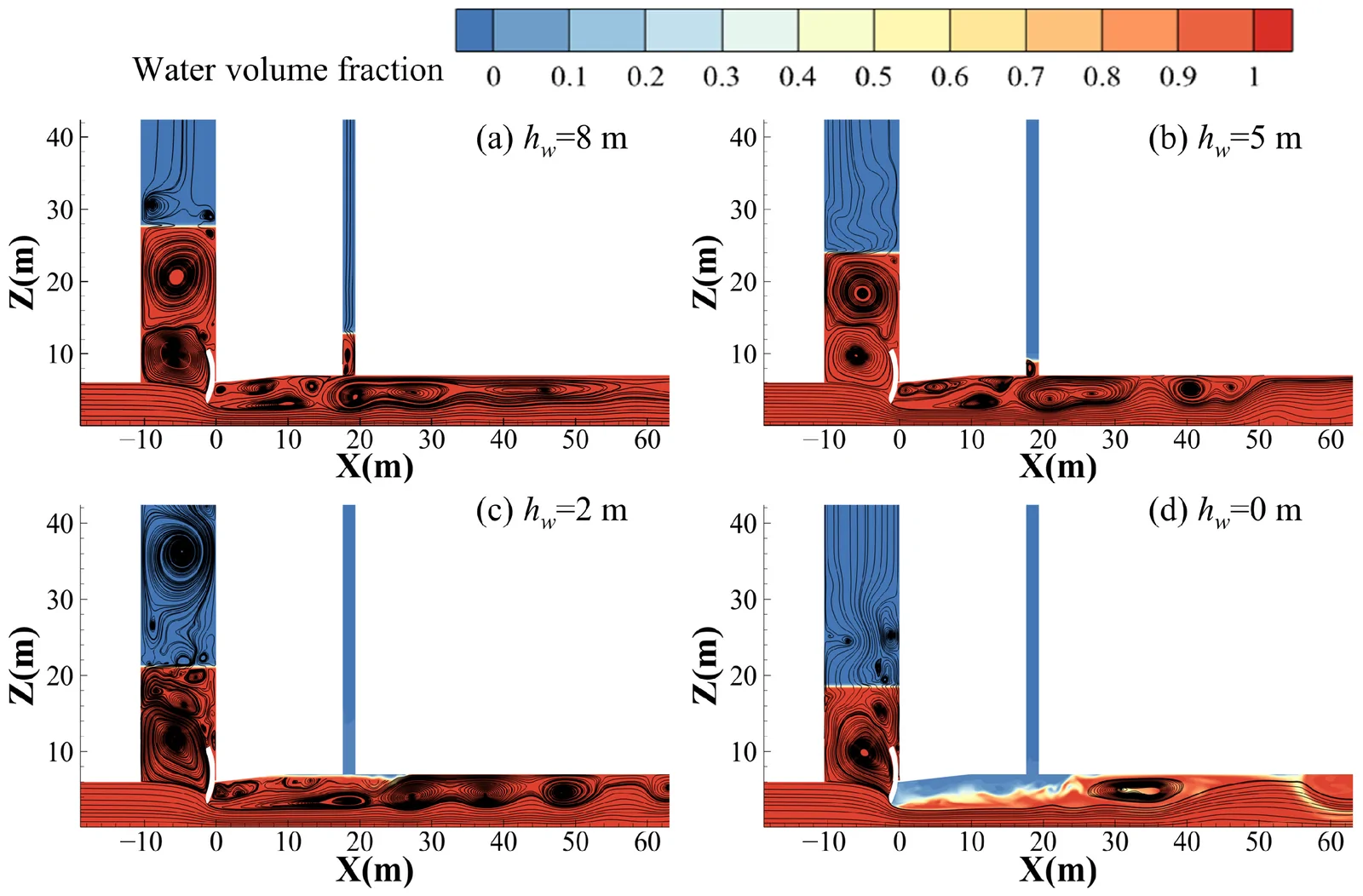
Water volume-fraction contours showing the post-valve flow regimes under different downstream-submergence depths at *v_i_* = 5 m/s: (**a**) *h_w_* = 8 m; (**b**) *h_w_* = 5 m; (**c**) *h_w_* = 2 m; (**d**) *h_w_* = 0 m.

Unless otherwise specified, all contour plots presented in the following analysis correspond to *t* = 200 s, which was selected as a representative late-stage instant following the initial transient development. As shown in Figure 7, at a fixed inlet velocity of *v_i_* = 5 m/s, the post-valve flow regime undergoes a progressive transition as the downstream-submergence depth *h_w_* decreases from 8 m to 0 m. The post-valve flow successively transitions from a water-sealed regime (Regime I) to a transition regime (Regime I–II), followed by a stable entrapped-air-pocket regime (Regime II) and, finally, a strongly unsteady hydraulic-jump-like regime (Regime III).

At *h_w_* = 8 m, the relatively high downstream water level exerts a pronounced backwater effect on the post-valve region (Figure 7a). The post-valve culvert remains pressurized, and a complete water seal is maintained at the bottom of the downstream shaft, preventing air from penetrating downward through the shaft into the culvert. The overall flow therefore exhibits a typical water-sealed regime (Regime I). At this stage, two large recirculation zones develop within the valve well, and the downstream flow field can be divided into the main-flow region, shear layer, and roller region. Although air intake through the shaft is not pronounced, local disturbances of the air–water interface induce several small-scale recirculation zones above the main flow, together with a larger recirculation structure beneath the shaft. These local interface disturbances and recirculation structures indicate that the post-valve flow remains hydrodynamically complex even though sustained shaft aeration does not occur.

At *h_w_* = 5 m, the reduced downstream water level weakens the backwater effect in the post-valve region, resulting in a pronounced depression of the free surface in the downstream shaft (Figure 7b). A small amount of air appears intermittently in the upper recirculation region. However, the water seal is not completely disrupted, and a continuous air-intake pathway between the downstream shaft and the post-valve culvert is not maintained. This condition therefore represents a transition regime (Regime I–II) between the water-sealed regime and the stable entrapped-air-pocket regime.

At *h_w_* = 2 m, the post-valve flow exhibits a stable entrapped-air-pocket regime (Regime II). As the downstream backwater effect further weakens, the water seal at the bottom of the downstream shaft is disrupted, allowing air to be continuously drawn into the post-valve culvert (Figure 7c). A coherent entrapped air pocket then forms along the culvert roof. Because the amplitude of the air–water interface oscillation remains relatively limited, the air-pocket morphology remains within a bounded fluctuating range despite persistent instantaneous oscillations.

At *h_w_* = 0 m, the post-valve flow develops into a strongly unsteady hydraulic-jump-like regime (Regime III). The confinement imposed by the downstream water level on the post-valve flow is largely removed, and air intake through the downstream shaft is markedly intensified, allowing a large amount of air to enter the post-valve culvert (Figure 7d). The entrapped air pocket along the culvert roof can no longer maintain a stable morphology. Instead, it undergoes pronounced interface overturning, breakup, and air–water remixing, together with strong wave-like propagation along the culvert. Compared with the stable entrapped-air-pocket regime at *h_w_* = 2 m, this case exhibits stronger temporal variability of the air–water interface and a higher degree of air–water mixing, indicating that the flow has entered a distinctly unsteady air–water two-phase regime. The resulting flow pattern resembles a hydraulic jump in free-surface flow, with intense air–water mixing and a large entrapped-air-pocket region formed near the downstream end of the jump.

Overall, the downstream-submergence depth (*h_w_*) is the key parameter governing the late-stage post-valve flow regime, as it controls the air-intake behavior of the downstream shaft and the resulting flow pattern in the post-valve culvert. At relatively large *h_w_*, the post-valve region is dominated by a water seal, and air intake through the shaft remains insignificant. As *h_w_* decreases from 5 m to 2 m, the flow changes from a transitional condition with intermittent air-pocket formation to a stable entrapped-air-pocket regime with a sustained air-intake pathway. This identifies *h_w_* = 2–5 m as the transition interval for sustained shaft aeration under the investigated conditions. Under extremely low submergence conditions, air intake through the downstream shaft is greatly enhanced, the air–water interface undergoes large-amplitude oscillations, and the flow eventually develops into a strongly unsteady hydraulic-jump-like regime.

#### 4.1.3. Temporal Evolution of Entrapped-Air-Pocket Volume

**Figure 8:**
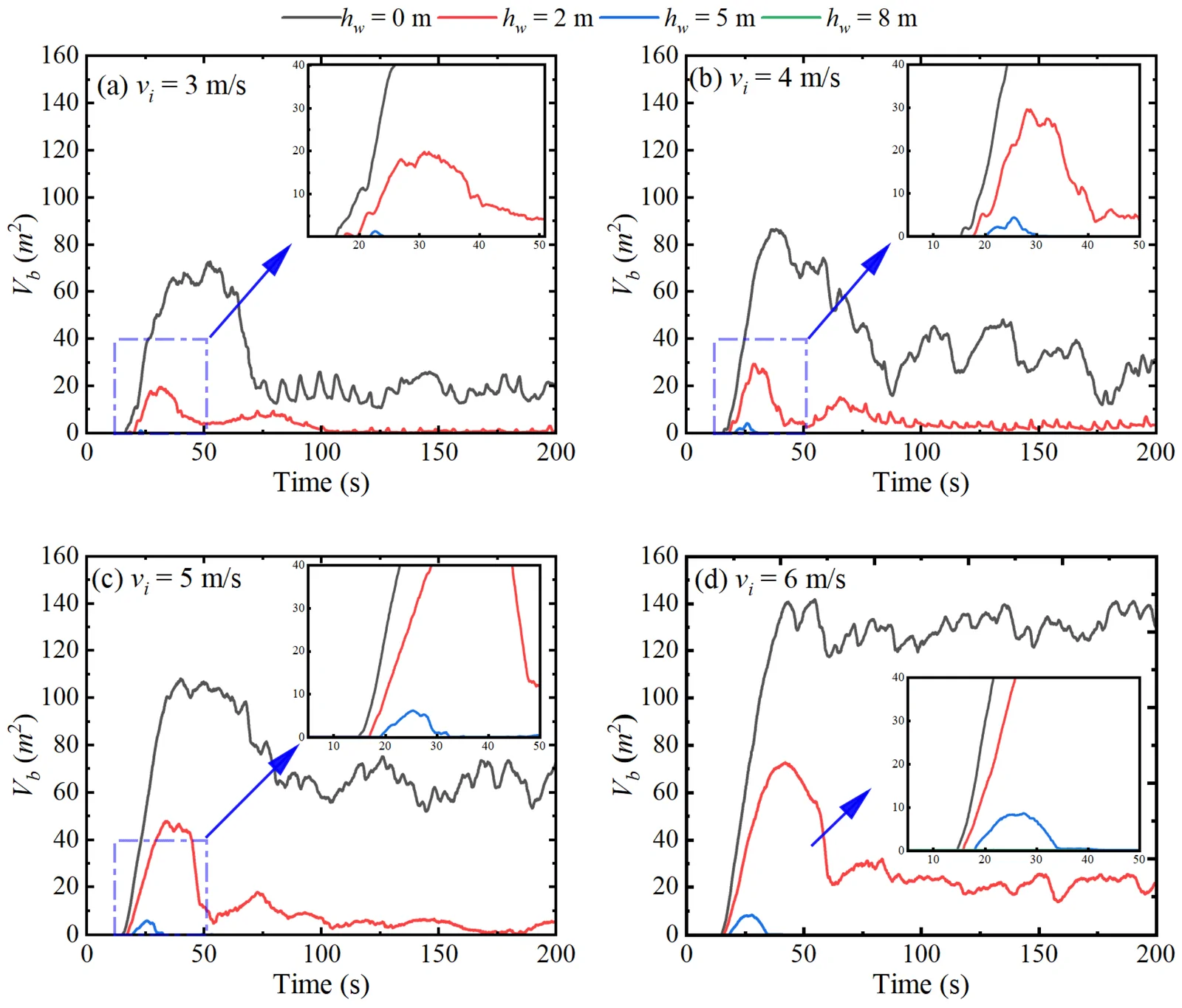
Temporal evolution of unit-width entrapped-air-pocket volume at valve opening *n* = 0.5 under different inlet velocities and downstream-submergence depths: (**a**) *v_i_* = 3 m/s; (**b**) *v_i_* = 4 m/s; (**c**) *v_i_* = 5 m/s; (**d**) *v_i_* = 6 m/s. For *h_w_* = 8 m, *V_b_* remains close to zero and therefore largely overlaps the horizontal axis at the present scale.

Figure 8 presents the temporal evolution of the unit-width entrapped-air-pocket volume *V_b_* under different combinations of inlet velocity *v_i_* = 3–6 m/s and downstream-submergence depth *h_w_*. The corresponding characteristic values are summarized in Table 4. Overall, *V_b_* exhibits a stage-dependent evolution process, including rapid growth, peak formation, decay and adjustment, and late-stage stabilization or sustained oscillation. The magnitude and persistence of *V_b_* vary markedly with *h_w_*, indicating that downstream-submergence depth (*h_w_*) governs the formation and maintenance of the entrapped air pocket.

At *h_w_* = 8 m, *V_b_* remains nearly zero for all inlet velocities. Only a weak transient response appears at the highest inlet velocity, and *V_b_*_,max_ remains within 0–0.026 m^2^. This indicates that the high downstream submergence maintains a stable water seal in the post-valve region and prevents sustained air-pocket formation. At *h_w_* = 5 m, *V_b_* shows a limited transient increase during the early stage and then rapidly decays toward zero. Although the transient peak becomes larger with increasing *v_i_*, the late-stage value *V_b_*_,s_ remains nearly zero. This behavior indicates that the water seal has been weakened and that local air entrainment can occur temporarily, but a continuous air-intake pathway has not yet been maintained. The corresponding flow condition represents a transition regime between the water-sealed regime and the stable entrapped-air-pocket regime.

When *h_w_* decreases to 2 m, *V_b_* increases markedly and a finite air-pocket volume persists after the initial peak. Both *V_b_*_,max_ and *V_b_*_,s_ increase compared with the cases of *h_w_* = 8 m and *h_w_* = 5 m, showing that the water seal at the shaft bottom has been disrupted and that a continuous air-intake pathway has formed between the downstream shaft and the culvert roof. Under this condition, the entrapped air pocket can be sustained along the post-valve culvert roof under the combined effects of shaft air supply, main-flow shear, and roof confinement. The post-valve flow therefore enters a stable entrapped-air-pocket regime. At *h_w_* = 0 m, *V_b_* reaches the highest level and continues to fluctuate strongly during the late stage. The entrapped air pocket can no longer maintain a quasi-stable morphology, but undergoes repeated interface overturning, breakup, and air–water remixing. The corresponding post-valve flow develops into a strongly unsteady hydraulic-jump-like regime. For the strongly unsteady *h_w_* = 0 m condition, *V*_*b*,s_ should therefore be interpreted as a late-stage statistical mean used for cross-condition comparison, rather than as a steady-state air-pocket volume.

The two characteristic indicators *V_b_*_,max_ and *V_b_*_,s_ further clarify the regime transition with decreasing downstream-submergence depth. *V_b_*_,max_ reflects the maximum short-term air-pocket expansion capacity, whereas *V_b_*_,s_ represents the late-stage air-pocket persistence. At *h_w_* = 8 m, both indicators remain nearly zero, corresponding to the water-sealed regime. At *h_w_* = 5 m, *V_b_*_,max_ becomes finite but *V_b_*_,s_ remains close to zero, indicating transient air-pocket formation without stable persistence. At *h_w_* = 2 m, the simultaneous increase in *V_b_*_,max_ and *V_b_*_,s_ confirms that a stable entrapped air pocket can be maintained in the post-valve culvert. At *h_w_* = 0 m, the further increase and strong fluctuation in both indicators indicate stronger air-pocket expansion and persistent air–water mixing under extremely low submergence. The contrasting responses at *h_w_* = 5 m and *h_w_* = 2 m therefore identify a downstream-submergence transition interval of *h_w_* = 2–5 m for the onset of sustained shaft aeration under the present geometry and fixed valve opening. Within this interval, the post-valve flow changes from transient air-pocket formation without stable retention to sustained air intake with a persistent entrapped air pocket. It should be emphasized that the quantitative boundaries of this transition interval are specific to the present geometric configuration and may shift with variations in shaft height and arrangement, as well as the culvert cross-section.

The inlet velocity mainly affects air-pocket evolution after aeration has been initiated. Under high-submergence conditions, increasing *v_i_* enhances local interface disturbance but does not create a stable entrapped air pocket because the water seal is still maintained. Once *h_w_* decreases to 2 m or 0 m, a continuous air-intake pathway is established, and increasing *v_i_* leads to larger *V_b_*_,max_ and *V_b_*_,s_. This result indicates that downstream-submergence depth determines whether shaft aeration and air-pocket persistence can occur, while inlet velocity mainly controls the intensity and temporal variability of the entrapped air pocket after the air-intake pathway has formed. This trend agrees with Deng [28], who found that increasing downstream submergence suppressed air intrusion and stabilized the post-valve flow.

**Figure 9:**
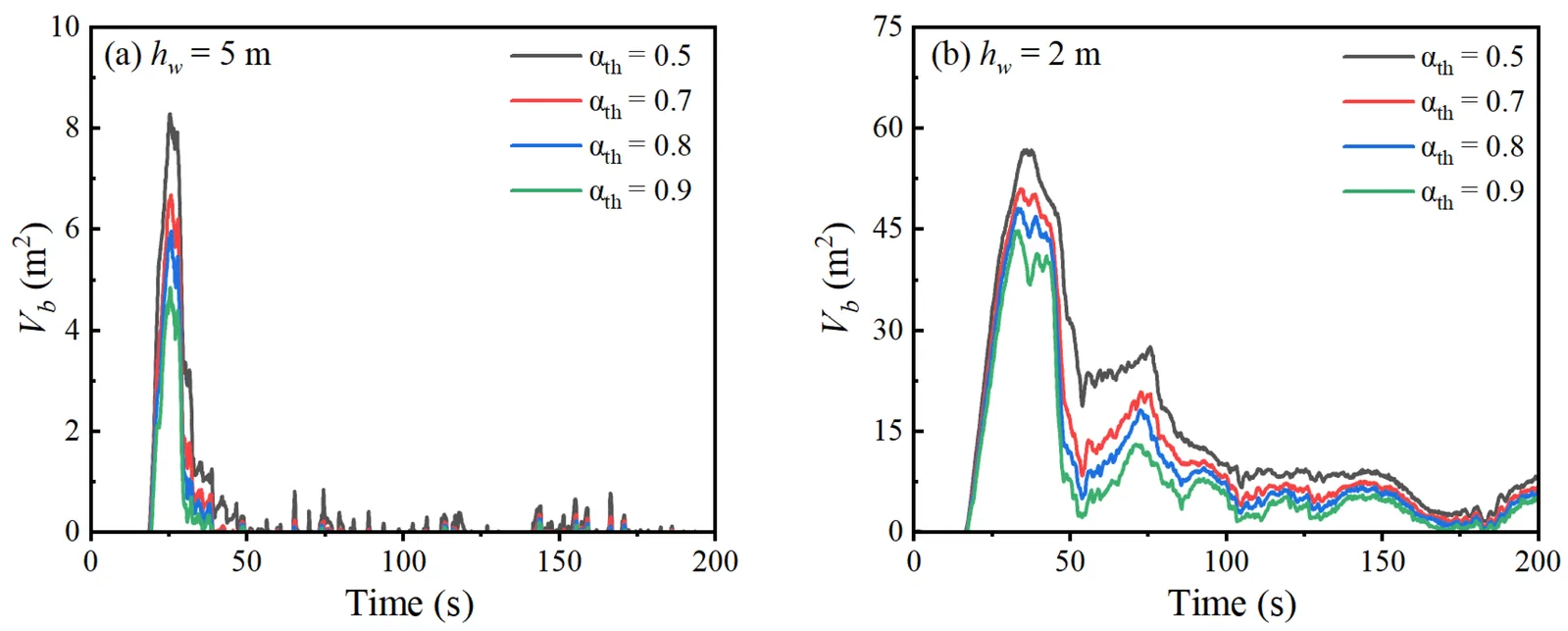
Sensitivity of the calculated entrapped-air-pocket volume to the air-volume-fraction threshold for representative cases: (**a**) *h_w_* = 5 m and (**b**) *h_w_* = 2 m (*v_i_* = 5 m/s, *n* = 0.5).

To assess the sensitivity of the flow-regime classification to the air-volume-fraction threshold, the air-pocket volume was recalculated for *h_w_* = 5 and 2 m at *v_i_* = 5 m/s and *n* = 0.5. As shown in Figure 9, the calculated air-pocket volume decreases systematically with increasing threshold because regions with lower local air volume fractions are progressively excluded. For *h_w_* = 5 m, the air pocket formed during the initial aeration stage rapidly decays, and no persistent air pocket is maintained during the later stage for *α*_th_ = 0.5–0.9. In contrast, for *h_w_* = 2 m, a finite air-pocket volume persists throughout the later stage under all tested thresholds. Therefore, although the absolute magnitude of the calculated air-pocket volume depends on the selected threshold, the qualitative distinction between transient air-pocket formation at *h_w_* = 5 m and sustained air-pocket retention at *h_w_* = 2 m remains unchanged. The threshold-sensitivity analysis therefore supports the classification of these two cases as the bounding states of the transition from transient to sustained shaft aeration.

### 4.2. Shaft Aeration and Pressure Response

Based on the above regime classification, the four cases at *v_i_* = 5 m/s were selected for further analysis of the pressure field, vortex structures, shear-layer characteristics, and pressure response under different downstream-submergence conditions.

#### 4.2.1. Spatial Pressure Distribution

**Figure 10:**
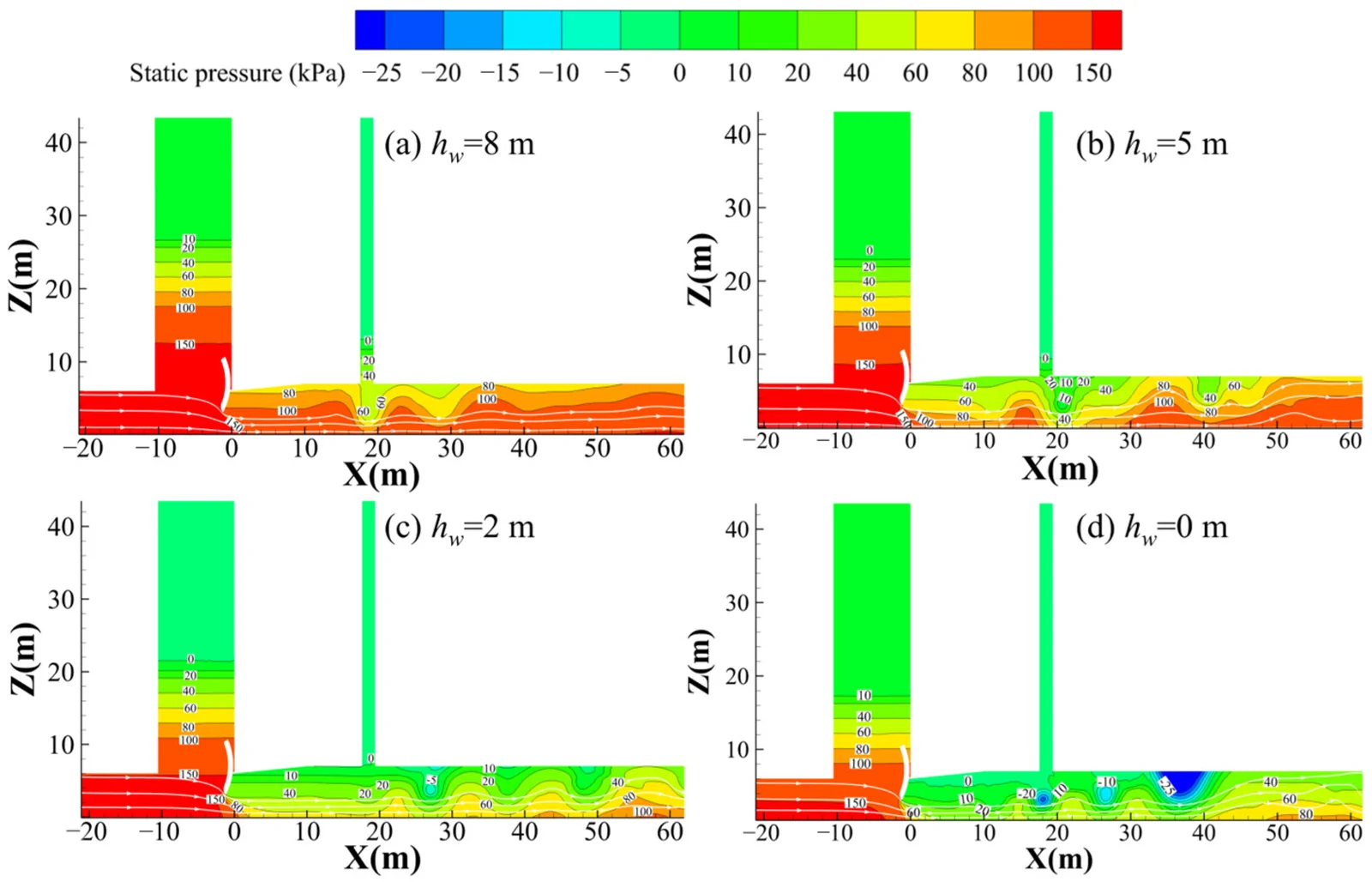
Static-pressure distributions in the post-valve region at valve opening *n* = 0.5 under different downstream-submergence depths at *v_i_* = 5 m/s: (**a**) *h_w_* = 8 m; (**b**) *h_w_* = 5 m; (**c**) *h_w_* = 2 m; (**d**) *h_w_* = 0 m.

To clarify the pressure-field basis for downstream-shaft aeration and entrapped-air-pocket formation, Figure 10 presents the static-pressure distributions in the post-valve region at *v_i_* = 5 m/s under different downstream-submergence depths. The low-pressure region is not uniformly distributed. It is mainly concentrated on the downstream side of the shaft, near the upper part of the post-valve recirculation region, and within the shear-layer mixing zone. These regions correspond well to the air-pocket distribution, indicating that local pressure depression provides the driving condition for air intake through the downstream shaft and air-pocket formation in the post-valve culvert.

As the downstream-submergence depth decreases, the low-pressure region near the culvert crown changes from isolated local patches into a more continuous low-pressure band extending downstream. At *h_w_* = 8 m and *h_w_* = 5 m, the low-pressure region remains limited in extent and lacks sufficient continuity, making it difficult to sustain shaft aeration. When *h_w_* decreases to 2 m, a relatively stable low-pressure band forms near the upper shear layer, providing persistent pressure conditions for maintaining the entrapped air pocket beneath the culvert roof. With *h_w_* further reduced to 0 m, the low-pressure region becomes more spatially fragmented and develops a multi-core undulating pattern. This indicates enhanced shear-layer mixing and stronger interfacial disturbance, corresponding to the strongly unsteady hydraulic-jump-like regime.

The pressure distribution identifies the low-pressure regions that promote shaft aeration, but it does not directly describe how entrapped air is transported and reorganized in the post-valve culvert. Therefore, the vortex structures are further examined using the Ω criterion as the primary identification method, with the *Q* and *λ*_2_ criteria additionally employed for cross-validation.

#### 4.2.2. Vortex and Shear-Layer Characteristics

To characterize the vortical structures associated with post-valve air-pocket evolution, the Ω criterion was adopted as the primary vortex-identification method [29]. The Ω-based vortex-identification method is defined as follows:

(14) $$\Omega =\frac{b}{a+b+\delta }$$

(15) $$a=\mathrm{trace}\left(A^{T}A\right)=\sum_{i=1}^{3}\sum_{j=1}^{3}{\left(A_{ij}\right)}^{2}$$

(16) $$b=\mathrm{trace}\left(B^{T}B\right)=\sum_{i=1}^{3}\sum_{j=1}^{3}{\left(B_{ij}\right)}^{2}$$

Here, ***A*** and ***B*** denote the strain-rate tensor and the vorticity, or rotation, tensor obtained from the decomposition of the velocity-gradient tensor, respectively. The tensor ***A*** is symmetric, whereas ***B*** is antisymmetric. If all components of ***B*** vanish, the flow is irrotational. The quantities *a* and *b* are the squared Frobenius norms of ***A*** and ***B***, respectively, and *δ* is a small positive number introduced to avoid division by zero in the computation. The Ω parameter represents the relative contribution of local rotation to the overall motion of a fluid element. Specifically, Ω = 0 corresponds to purely deformational motion, whereas Ω = 1 represents ideal rigid-body rotation. Regions with relatively large Ω values can therefore be identified as rotation-dominated zones; the closer Ω is to unity, the more closely the local flow approaches pure rigid-body rotation.

To assess whether the identified dominant vortex structures depend on the selected identification method, the *Q* and *λ*_2_ criteria were additionally introduced for cross-validation [30]. Based on the strain-rate tensor ***A*** and rotation tensor ***B*** defined above, the *Q* criterion is expressed as:

(17) $$Q=\frac{1}{2}\left({\left\|B\right\|}_{F}^{2}-{\left\|A\right\|}_{F}^{2}\right)=\frac{1}{2}(b-a)$$

where *Q* > 0 denotes a rotation-dominated region [31]. The λ_2_ criterion is based on the eigenvalues of the tensor ***A***^2^ + ***B***^2^. When the eigenvalues are ordered as *λ*_1_ ≥ *λ*_2_ ≥ *λ*_3_, a vortex core is identified by λ_2_ < 0 [32].

**Figure 11:**
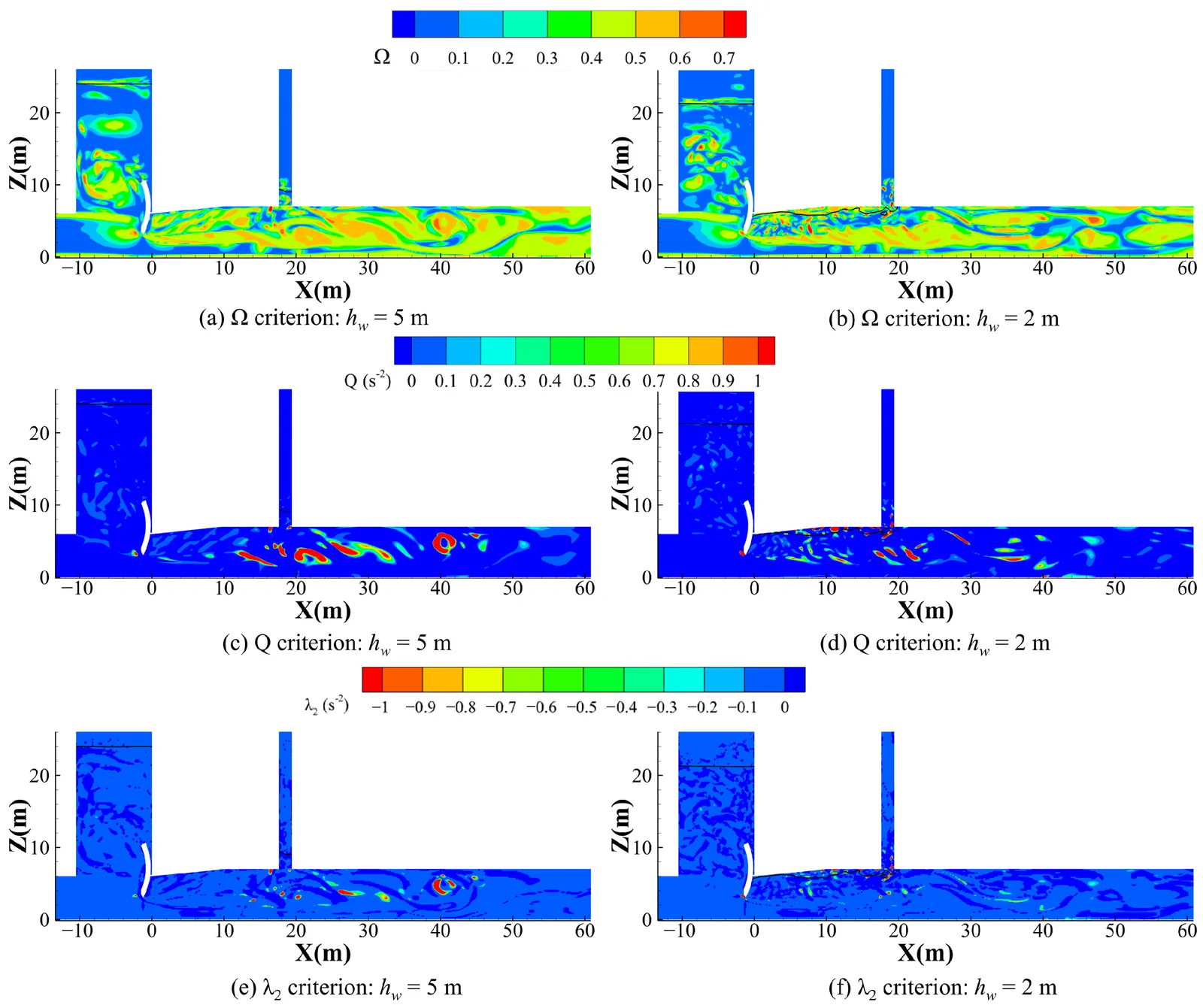
Cross-validation of vortex structures identified using different vortex-identification criteria: (**a**,**b**) Ω criterion; (**c**,**d**) *Q* criterion; and (**e**,**f**) *λ*_2_ criterion. The left and right columns correspond to *h_w_* = 5 and 2 m, respectively (*v_i_* = 5 m/s, *n* = 0.5).

For cross-validation, the *h_w_* = 5 and 2 m cases were compared using the Ω, *Q*, and *λ*_2_ criteria, with *v_i_* = 5 m/s and *n* = 0.5. For visualization clarity, the display ranges were limited to Ω = 0–0.7, *Q* = 0–1 s^−2^, and *λ*_2_ = −1–0 s^−2^, respectively. These ranges were used only to enhance the visibility of the vortex structures. As shown in Figure 11, the three criteria identify generally consistent locations and spatial distributions of the dominant vortex structures. The Ω and *Q* criteria exhibit similar overall vortex patterns, whereas the λ_2_ criterion identifies relatively more localized vortex-core regions. Despite differences in the detailed vortex boundaries, the principal vortex structures in the post-valve region are consistently captured by the three criteria, supporting the robustness of the Ω-based vortex identification used in the following analysis.

**Figure 12:**
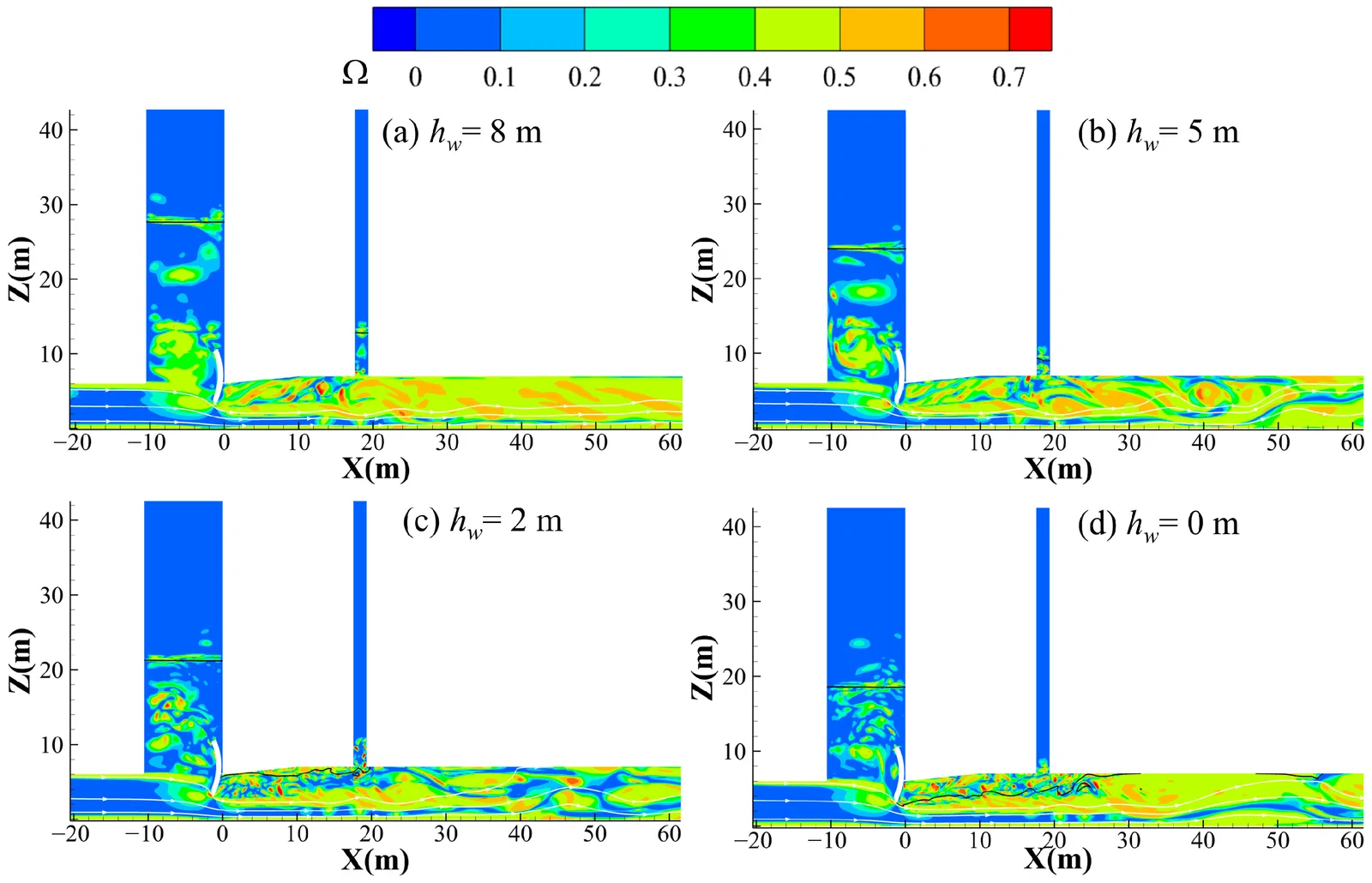
Vortex structures identified using the Ω criterion and corresponding air–water interfaces under different downstream-submergence depths at *v_i_* = 5 m/s: (**a**) *h_w_* = 8 m, (**b**) *h_w_* = 5 m, (**c**) *h_w_* = 2 m, (**d**) *h_w_* = 0 m.

The vortex structures under the four downstream-submergence conditions are further compared using the Ω criterion, as shown in Figure 12. The black lines denote the air–water interface. The vortex structures are mainly concentrated at the shaft–culvert junction, near the upper downstream side of the shaft, within the post-valve shear layer, and along the upper part of the recirculation region. These regions show clear spatial correspondence with the entrapped-air-pocket distribution, indicating that entrapped air is not randomly distributed in the culvert. Instead, it is preferentially transported and reorganized within the interfacial mixing region controlled by the shear layer [33].

At *h_w_* = 8 m, the vortex structures are largely confined to the shaft and near-valve region. High-intensity vortices in the upper shear layer of the main flow remain limited in extent, indicating that local recirculation and interface disturbance are insufficient to sustain continuous shaft aeration. When *h_w_* decreases to 5 m, vortex activity becomes more concentrated within the downstream shear layer, and the flow approaches a transition regime between the water-sealed regime and the stable entrapped-air-pocket regime. As *h_w_* further decreases to 2 m, relatively continuous coherent rotational structures develop within the downstream shear layer and coincide with the boundary of the stable entrapped air pocket. This correspondence shows that the formation and maintenance of the stable entrapped air pocket are closely associated with persistent vortex activity in the shear layer. At *h_w_* = 0 m, the post-valve vortex structures become more fragmented and spatially reorganized, corresponding to intensified interfacial mixing and strongly unsteady air-pocket breakup.

To further quantify the vortex characteristics under different downstream-submergence conditions, the vortex–area ratio Ω*_p_* and mean vortex intensity Ω_0.5_ were evaluated within the fixed post-valve culvert region [34]. They are defined as:

(18) $$\Omega_{p}=S_{0.5}/S_{c}$$

(19) $$\Omega_{0.5}=\frac{1}{S_{0.5}}\int_{S_{0.5}}\Omega \;dS$$

where *S*_0.5_ is the area satisfying Ω > 0.5 within the predefined post-valve statistical region, and *S_c_* is the fixed statistical area of the post-valve culvert. The same statistical region was used for all cases, with *S_c_* = 1374.95 m^2^. *S*_0.5_ was obtained by area integration over the region satisfying Ω > 0.5.

As shown in Table 5, Ω*_p_* decreases from 0.0514 at *h_w_* = 8 m to 0.0199 at *h_w_* = 2 m and then slightly increases to 0.0249 at *h_w_* = 0 m. In contrast, Ω_0.5_ varies only slightly from 0.52 to 0.54 among the four conditions. These results indicate that downstream submergence has a more pronounced effect on the spatial extent of the identified vortex regions than on their mean Ω intensity.

To quantitatively characterize the post-valve shear layer, velocity profiles at *x* = 20 m were analyzed over *t* = 100–200 s using 21 instantaneous flow fields sampled at 5 s intervals. The characteristic shear intensity *Gs* was defined as the magnitude of the strongest negative time-averaged vertical velocity gradient within the upper shear region of the post-valve jet. The resolved temporal velocity fluctuation amplitude was further evaluated as

(20) $${u^{\prime }}_{\mathrm{rms}}(z)=\sqrt{\frac{1}{N}\sum_{i=1}^{N}{\left[U_{x}(z,t_{i})-{\bar{U}}_{x}(z)\right]}^{2}}$$

where ${u^{\prime }}_{\mathrm{rms}}(z)$ is the resolved temporal velocity fluctuation amplitude at vertical position *z*; *U_x_* (*z*, *t_i_*) is the instantaneous streamwise velocity at the *i*th sampling instant *t_i_*; *U_x_*(*z*) is the time-averaged streamwise velocity; and *N* is the number of sampled instantaneous flow fields (*N* = 21).

**Figure 13:**
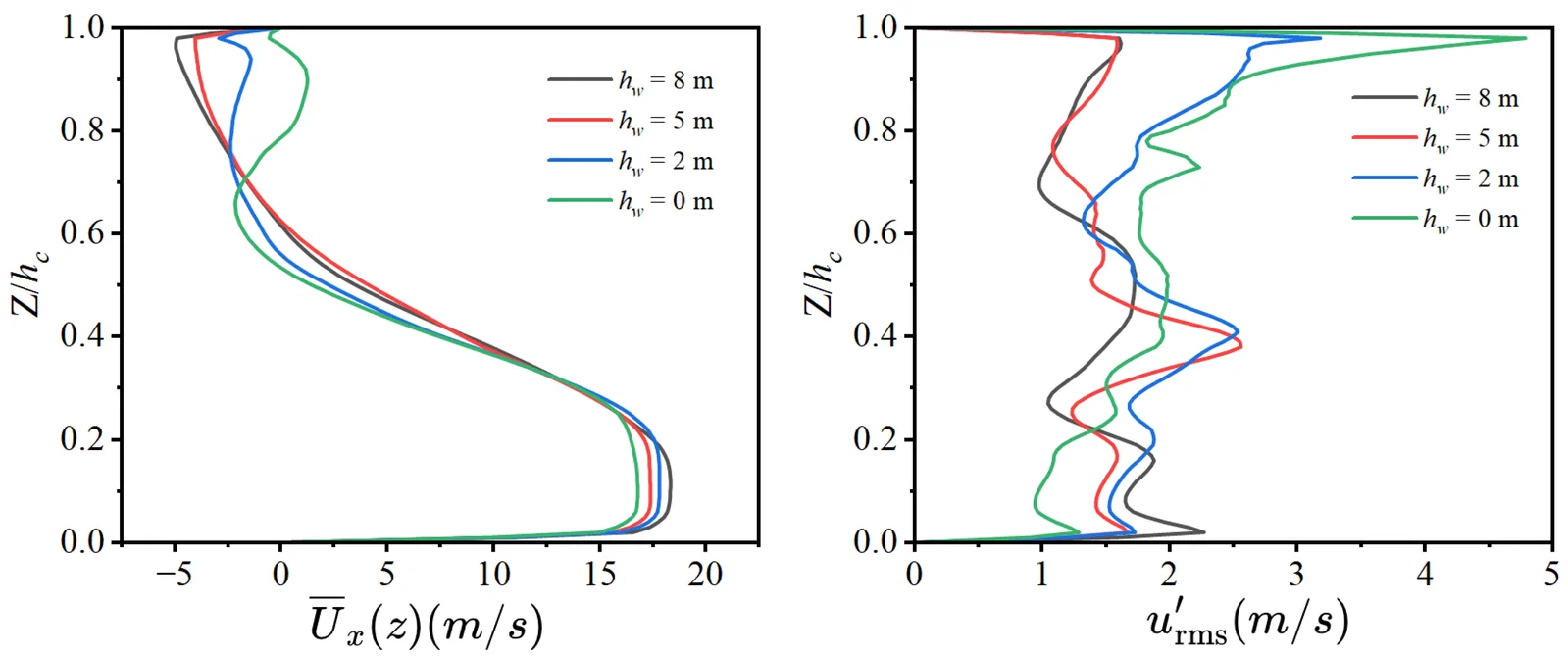
Time-averaged streamwise velocity and resolved temporal velocity fluctuation profiles at *x* = 20 m under different downstream-submergence depths: (**a**) $\bar{U_{x}}\left(z\right)$; (**b**) ${u^{\prime }}_{\mathrm{rms}}$ (*v_i_* = 5 m/s, *n* = 0.5; statistics over *t* = 100–200 s).

As shown in Figure 13 and Table 6, the characteristic shear intensity *G_s_* remains approximately 8.0 s^−1^ at *h_w_* = 8 and 5 m, and increases to 9.91 and 10.35 s^−1^ at *h_w_* = 2 and 0 m, respectively. In particular, the transition from *h_w_* = 5 to 2 m is accompanied by an approximately 24% increase in *G_s_*, together with an increase in *V_b_*_,max_ from 6.102 to 48.162 m^2^. The resolved temporal velocity fluctuation amplitude reaches its maximum value of 2.33 m/s at *h_w_* = 2 m. However, neither *G_s_* nor ${u^{\prime }}_{\mathrm{rms}}$ varies proportionally with *V_b_*_,max_. For example, from *h_w_* = 2 to 0 m, *G_s_* increases only slightly from 9.91 to 10.35 s^−1^, whereas *V_b_*_,max_ increases from 48.162 to 108.363 m^2^. These results indicate that enhanced post-valve shear is closely associated with sustained shaft aeration, but shear-layer strengthening alone does not determine entrapped-air-pocket development.

The quantitative shear-layer characteristics are consistent with the vortex-structure evolution discussed above and with the shear-flow-instability mechanism reported in Ref. [25], where air-pocket entrapment was interpreted as a Kelvin–Helmholtz-type instability associated with relative motion between the air and water phases. In the present configuration, the post-valve jet interacts with the upper recirculation region to form a persistent shear layer. The increase in *G_s_* from *h_w_* = 5 m to *h_w_* = 2 m provides quantitative support for the involvement of this shear layer in the transition from transient to sustained shaft aeration, whereas the non-proportional variation in *G_s_* and *V_b_*_,max_ indicates that shear-layer instability is not the sole controlling mechanism. Local pressure depression provides the driving condition for air intake, the shear layer promotes interfacial deformation and instability, and coherent vortical structures facilitate the downstream transport and reorganization of entrapped air. Downstream-shaft aeration is therefore interpreted as being associated with the combined effects of local pressure depression, jet-induced shear, vortex-mediated air transport, and air supply through the shaft. This interpretation is consistent with previous studies linking the downstream crown-pressure drop to the recirculation region [26] and showing that introduced air can be transported into shear-flow and vortex-core regions [11].

#### 4.2.3. Pressure Response of the Shaft–Post-Valve System

**Figure 14:**
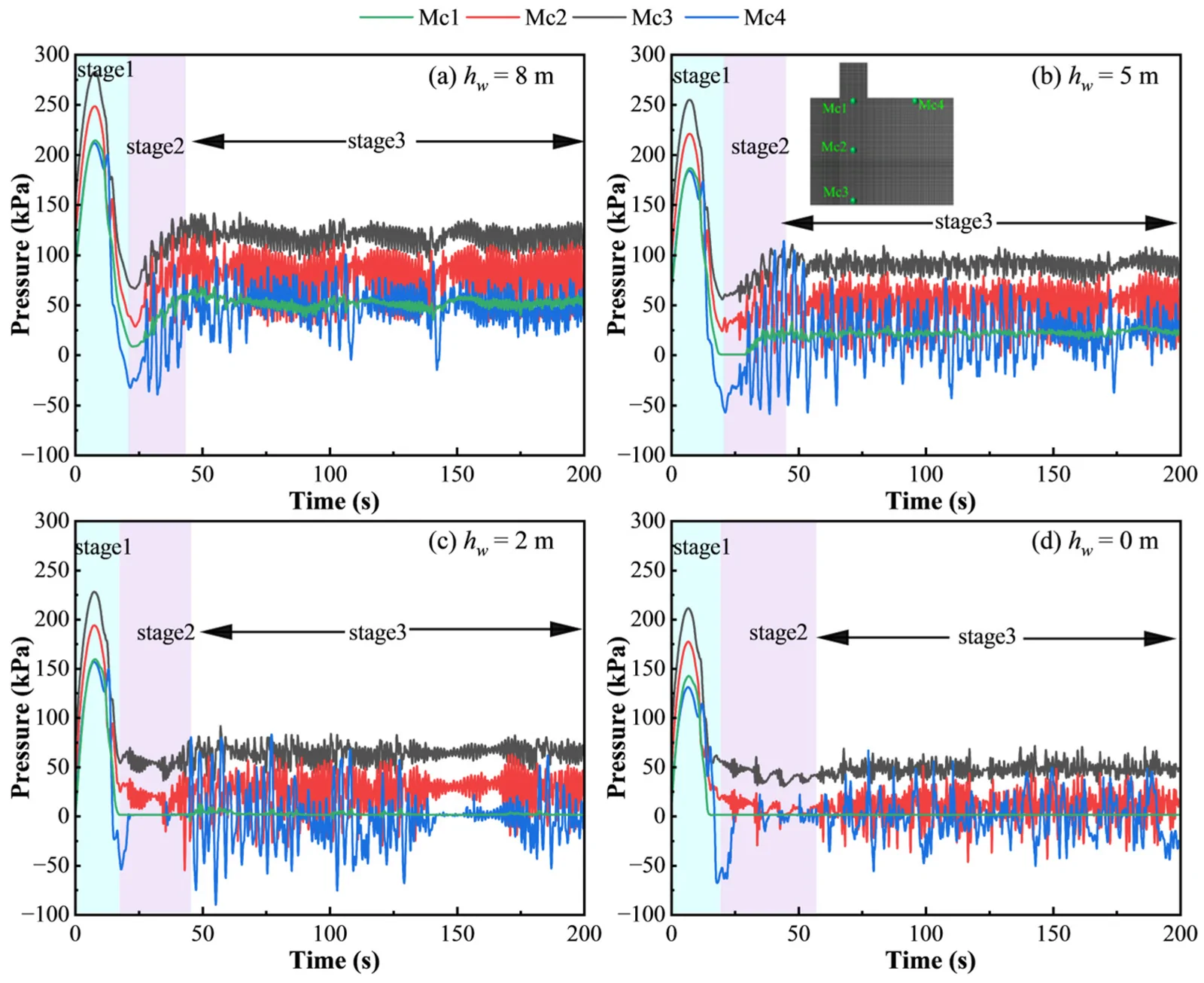
Pressure time histories at representative monitoring points in the near-shaft region at *v_i_* = 5 m/s: (**a**) *h_w_* = 8 m, (**b**) *h_w_* = 5 m, (**c**) *h_w_* = 2 m, (**d**) *h_w_* = 0 m.

**Figure 15:**
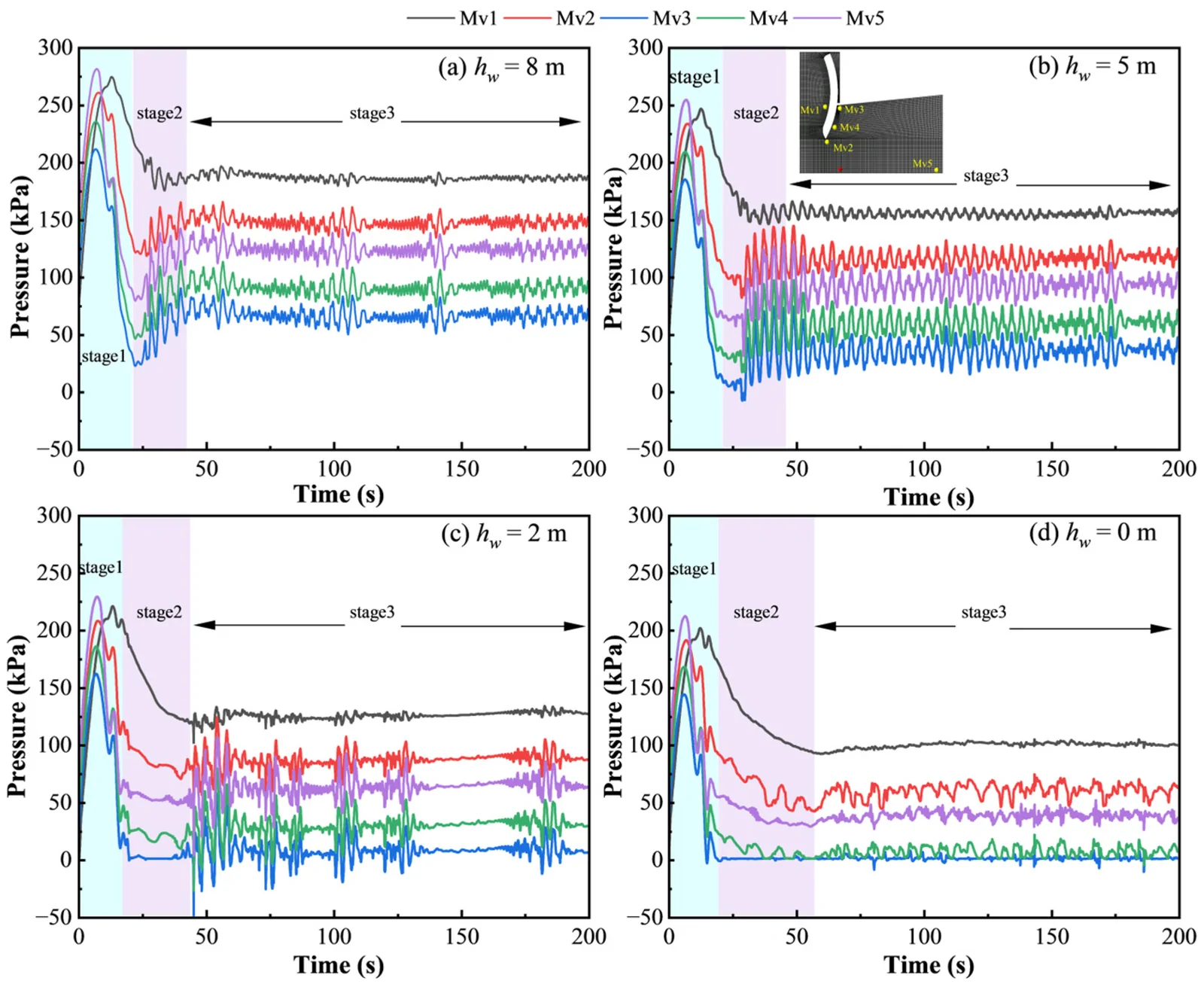
Pressure time histories at representative monitoring points in the near-valve region at *v_i_* = 5 m/s: (**a**) *h_w_* = 8 m, (**b**) *h_w_* = 5 m, (**c**) *h_w_* = 2 m, (**d**) *h_w_* = 0 m. The red dot indicates the coordinate origin defined in Figure 2.

To characterize the temporal pressure response after the onset of downstream-shaft aeration, pressure histories at representative monitoring points in the downstream shaft and near the valve were compared at a fixed inlet velocity of *v_i_* = 5 m/s. Figure 14 shows the pressure histories in the near-shaft region, and Figure 15 shows those in the near-valve region. The pressure response of the shaft–post-valve system can be divided into three stages: jet establishment, low-pressure development, and late-stage pressure pulsation. As the downstream-submergence depth *h_w_* decreases from 8 m to 0 m, the initial pressure peak becomes lower, the onset of negative pressure occurs earlier, and the negative-pressure duration becomes longer. This trend indicates that reduced downstream submergence weakens the hydraulic confinement of the post-valve flow and promotes earlier air intake through the downstream shaft.

The spatial difference among monitoring points reflects the development of the post-valve low-pressure region. In the downstream shaft, the shaft-bottom pressure remains relatively high, whereas the downstream-side point shows the earliest and strongest pressure drop. Near the valve, the upstream face maintains higher pressure than the downstream face, and the pressure reduction on the downstream face becomes more pronounced as *h_w_* decreases. The low-pressure region therefore not only drives shaft aeration but also increases the local pressure difference across the valve.

The pressure response is strongly regime-dependent. At *h_w_* = 8 m, the water-sealed post-valve region is dominated by positive pressure and limited oscillation. At *h_w_* = 5 m, transient negative pressure occurs, but the pressure fluctuation is insufficient to maintain a stable entrapped air pocket. At *h_w_* = 2 m, the stable entrapped air pocket repeatedly interacts with the high-speed jet and roof-confined shear layer, producing the strongest alternating positive- and negative-pressure fluctuations. When *h_w_* decreases to 0 m, the negative-pressure duration is prolonged, while the late-stage oscillation amplitude decreases. The flow is then characterized mainly by prolonged negative-pressure exposure and intense air–water mixing rather than by the strongest pressure pulsation.

The indicators at Mc4 support this interpretation, as summarized in Table 7. All pressure values reported herein are gauge pressures. During 100–200 s, the minimum pressure decreases from −15.28 kPa at *h_w_* = 8 m to −77.02 kPa at *h_w_* = 2 m, while *p*_RMS_ reaches its maximum value of 22.04 kPa at *h_w_* = 2 m. This indicates that the stable entrapped-air-pocket regime is associated with the strongest localized pressure oscillation. By contrast, the *h_w_* = 0 m case has the longest negative-pressure duration of 67.30 s, but its *p*_RMS_ is only 8.22 kPa, indicating prolonged negative-pressure exposure rather than maximum pulsation.

These results show that the most unfavorable hydraulic condition cannot be judged only from the lowest downstream water level. At *v_i_* = 5 m/s, the *h_w_* = 2 m case is more unfavorable in terms of localized pressure depression and pressure fluctuations, because a stable entrapped air pocket persists and couples with the post-valve jet and shear-layer vortices. The *h_w_* = 0 m case is characterized by a longer negative-pressure duration and stronger air–water mixing. Therefore, the pressure risk should be evaluated from the combined response of pressure level, pressure fluctuation intensity, negative-pressure duration, and entrapped-air-pocket evolution.

## 5. Conclusions

The downstream-shaft aeration and entrapped-air-pocket evolution downstream of a navigation-lock valve were investigated using a three-dimensional VOF model. The main conclusions are as follows.

(1) Downstream-submergence depth is the dominant factor controlling downstream-shaft aeration and entrapped-air-pocket formation. With decreasing *h_w_*, the post-valve flow regime changes from a water-sealed regime to a transition regime, then to a stable entrapped-air-pocket regime and a strongly unsteady hydraulic-jump-like regime. For the present geometry and fixed valve opening, the transition to sustained shaft aeration is bracketed between *h_w_* = 5 m and *h_w_* = 2 m.

(2) The inlet velocity mainly affects the aeration intensity after a continuous air-intake pathway has been established. Under high-submergence conditions, increasing *v*_i_ enhances local interface disturbance but does not maintain a stable entrapped air pocket. Once *h_w_* decreases to 2 m or 0 m, a continuous air-intake pathway forms, and larger *v*_i_ leads to greater unit-width entrapped-air-pocket volume *V_b_* and stronger temporal variability of the air pocket.

(3) Quantitative shear-layer analysis shows that *G_s_* increases from about 8.0 s^−1^ at *h_w_* = 8 and 5 m to 9.91 and 10.35 s^−1^ at *h_w_* = 2 and 0 m, respectively, while ${u^{\prime }}_{\mathrm{rms}}$ reaches 2.33 m/s at *h_w_* = 2 m. Together with the Ω-based vortex structures, these results indicate that enhanced post-valve shear is closely associated with sustained shaft aeration and air-pocket evolution. The non-proportional variation in *G_s_* and *V_b_*_,max_ further suggests that air-pocket development reflects the coupled effects of negative-pressure-driven air intake, shear-layer development, and vortex-mediated air transport.

(4) The pressure response is closely related to the corresponding air-pocket regime. At *v_i_* = 5 m/s, the *h_w_* = 2 m condition is more unfavorable in terms of localized pressure depression and pressure fluctuations, accompanied by repeated interactions between the air pocket, the high-speed jet, and coherent vortical structures within the shear layer. At *h_w_* = 0 m, the post-valve flow develops into a strongly unsteady hydraulic-jump-like regime characterized by a longer negative-pressure duration and stronger air–water mixing. These results indicate that the most unfavorable hydraulic condition should be evaluated from the combined response of pressure level, pressure fluctuation intensity, negative-pressure duration, and entrapped-air-pocket evolution, rather than from downstream water level alone.

The reverse tainter valve and post-valve culvert configuration considered in this study are representative of commonly used configurations in navigation-lock water conveyance systems, and the selected relative valve opening of *n* = 0.5 corresponds to a relatively unfavorable post-valve flow condition. Therefore, the identified flow-regime evolution and aeration mechanisms have practical relevance. However, the specific boundaries of the *h_w_* = 2–5 m transition interval may vary with valve opening, shaft configuration, and culvert geometry. Further studies should address these effects together with full-width transverse flow and prototype-scale validation.

## Figures and Tables

**Table 1:** Constants used in the RNG k–ε turbulence model.

Parameter	Value
$\eta_{0}$	4.38
β	0.012
$C_{\mu }$	0.0845
$C_{1\varepsilon }$	1.42
$C_{2\varepsilon }$	1.68
$\sigma_{k}$	0.7179
$\sigma_{\varepsilon }$	0.7179

**Table 2:** Summary of computational cases.

Parameter	Symbol	Value
Valve opening	n	0.5
Inlet velocity	$v_{i}$	3, 4, 5, 6 m/s
Downstream submergence	$h_{w}$	8, 5, 2, 0 m
Simulation duration	*T*	200 s

**Table 3:** Grid independence verification.

Grid	Cell Numbers	$\bar{v}$ (m/s)	$v_{\max}$ (m/s)	Relative Error ^1^ (%)
Coarse	512,190	12.42	14.87	8.72
Medium	1,116,425	13.54	16.16	0.80
Fine	1,988,465	13.59	16.29	--

^1^ Relative Error denotes the relative difference in *v*_max_ with respect to the fine-grid result.

**Table 4:** Summary of unit-width entrapped-air-pocket volume and corresponding post-valve flow regimes.

$h_{w}$ (m)	$\mathrm{Range}\;\mathrm{of}\;V_{b,\max}$ (m^2^)	$\mathrm{Mean}\;V_{b,\max}$ (m^2^)	$\mathrm{Range}\;\mathrm{of}\;V_{b,s}$ (m^2^)	$\mathrm{Mean}\;V_{b,s}$ (m^2^)	Flow Regime
8	0.000–0.026	0.006	0.000–0.000	0.000	Regime I
5	1.316–8.532	5.084	0.000–0.001	0.000	Regime I–II
2	19.762–72.898	42.586	0.969–21.593	7.448	Regime II
0	72.951–141.962	102.508	18.468–131.906	61.860	Regime III

Note: *V_b_*_,max_ denotes the maximum entrapped-air-pocket volume during *t* = 0–200 s, and *V_b_*_,s_ denotes the mean entrapped-air-pocket volume over *t* = 100–200 s. The reported ranges summarize the variation across *v_i_* = 3–6 m/s, while “Mean” denotes the arithmetic mean of the corresponding values across these four inlet velocities.

**Table 5:** Quantitative vortex characteristics at 200 s under different downstream-submergence conditions (*v_i_* = 5 m/s, *n* = 0.5).

$h_{w}$ (m)	*S*_0.5_ (m^2^)	Ω_*p*_	Ω_0.5_
8	70.68	0.0514	0.52
5	52.64	0.0383	0.53
2	27.34	0.0199	0.54
0	34.27	0.0249	0.54

**Table 6:** Quantitative shear-layer characteristics and corresponding maximum unit-width entrapped-air-pocket volume under different downstream-submergence depths (*v_i_* = 5 m/s, *n* = 0.5).

$h_{w}$ (m)	$G_{s}$ (s^−1^)	${u^{\prime }}_{\mathrm{rms}}$ (m/s)	$V_{b,\max}$ (m^2^)	Flow Regime
8	8.01	1.60	0	Regime I
5	7.98	2.16	6.102	Regime I–II
2	9.91	2.33	48.162	Regime II
0	10.35	1.88	108.363	Regime III

Note: *G_s_* denotes the characteristic shear intensity; ${u^{\prime }}_{\mathrm{rms}}$ denotes the resolved temporal velocity fluctuation amplitude at the characteristic shear-layer location; and *V_b_*_,max_ denotes the maximum unit-width entrapped-air-pocket volume during *t* = 0–200 s.

**Table 7:** Pressure-response indicators at Mc4 under different downstream-submergence depths (*v_i_* = 5 m/s, *n* = 0.5).

$h_{w}$ (m)	$p_{\min}$ (kPa)	$p_{\mathrm{RMS}}$ (kPa)	$t_{neg}$ (s)	Flow Regime
8	−15.28	17.32	1.70	Regime I
5	−43.74	21.74	19.05	Regime I–II
2	−77.02	22.04	61.70	Regime II
0	−39.80	8.22	67.30	Regime III

Note: *p*_min_ denotes the minimum pressure at Mc4, *p*_RMS_ denotes the root-mean-square value of the pressure fluctuation relative to the time-averaged pressure, and *t*_neg_ denotes the cumulative duration of negative pressure during 100–200 s. Regime I, Regime II, and Regime III represent the water-sealed regime, stable entrapped-air-pocket regime, and strongly unsteady hydraulic-jump-like regime, respectively. Regime I–II denotes the transition regime between Regime I and Regime II.

## Data Availability

The original contributions presented in this study are included in the article. The data supporting the findings of this study are available from the corresponding author upon reasonable request.
